# A Review on the General Cheese Processing Technology, Flavor Biochemical Pathways and the Influence of Yeasts in Cheese

**DOI:** 10.3389/fmicb.2021.703284

**Published:** 2021-07-29

**Authors:** Xiaochun Zheng, Xuewei Shi, Bin Wang

**Affiliations:** Food College, Shihezi University, Shihezi, China

**Keywords:** cheese, flavor compounds, general craftsmanship, yeast, biochemical pathways

## Abstract

Cheese has a long history and this naturally fermented dairy product contains a range of distinctive flavors. Microorganisms in variety cheeses are an essential component and play important roles during both cheese production and ripening. However, cheeses from different countries are still handmade, the processing technology is diverse, the microbial community structure is complex and the cheese flavor fluctuates greatly. Therefore, studying the general processing technology and relationship between microbial structure and flavor formation in cheese is the key to solving the unstable quality and standardized production of cheese flavor on basis of maintaining the flavor of cheese. This paper reviews the research progress on the general processing technology and key control points of natural cheese, the biochemical pathways for production of flavor compounds in cheeses, the diversity and the role of yeasts in cheese. Combined with the development of modern detection technology, the evolution of microbial structure, population evolution and flavor correlation in cheese from different countries was analyzed, which is of great significance for the search for core functional yeast microorganisms and the industrialization prospect of traditional fermented cheese.

## Introduction

Cheese is an ancient traditional fresh or fermented dairy product with a long history of production. Cheese making originated in various West Asian countries about 8,000 years ago (Sandine and Elliker, 1970); it is traditionally called “milk pimples” by Mongolian, Kazak, and other nomadic people in northwestern China (Zheng et al., 2021). Cheese is made by curdling milk, cream, or partially skimmed buttermilk from cow or goat or a mixture of these products and then separating the whey. In general, it is prepared by adding an appropriate amount of lactic acid bacteria (LAB) starter along with rennet to milk, the fermentation by which transforms the milk proteins (mainly casein), carbohydrates, and fats. Next, the whey is removed and the remaining product is ripened for a certain period. LAB may not be added for making some cheeses.

Fermented dairy products with high nutritional value are considered “the pearls in the crown of the dairy industry” (Kourkoutas et al., 2006). With the improvement of the quality of life, the people’s dietary needs and demands have been changing in terms of quantity and quality. Furthermore, the lactose content in cheese is low, and its consumption is thus highly suitable for people with lactose intolerance (Monti et al., 2017). Currently, nearly 130 countries and regions produce various cheese, and the total global cheese production measures nearly 2,000 × 10^4^ tons, with the European Union countries (e.g., Netherlands and Germany) being the largest cheese exporters worldwide. From the development perspective of the global dairy industry, cheese is a very important dairy product, but it has not yet become an independent industry in China. At present, cheese production in developing countries remains in its infancy, with the most individuals from these countries being unfamiliar with cheese. Simultaneously, dairy processing companies face funds and technologies limitations. Thus far, no cheese has been widely accepted. Therefore, the study of cheese is particularly urgent (Kourkoutas et al., 2006; El Sheikha, 2018). With the development of dairy industry in various countries, the vigorous development of the cheese industry has become the focus of attention in recent years.

Globally, there is a lack of an authoritative cheese-making process, which can be used for cheese production followed in any country – China or abroad. This is due to differences in regions, production methods, and available raw materials. The earliest method of producing cheese in the world was to carry the milk to the animal’s internal and the milk was fermented into cheese by constant oscillation during the migration. Cheese is made differently in different regions. For instance, for making cheddar cheese in southwest England, the raw material is sterilized and cooled; then, the fermenting agent, calcium chloride, and rennet are added to ferment the curd (Lawrence et al., 2004). After 30–40 min, the formed clot is cut into 5-mm sized pieces, which are then allowed to stand for 15 min and stirred for another 5–10 min. The clot is then turned over and stacked, broken, salted, molded, and pressed. The pressed cheese is put into a fermentation room for fermenting and ripening after the cloth is changed (Banks et al., 1989; Azarnia et al., 2006). In contrast, the milk used to make Parmesan cheese is collected in two separate steps, where the overnight milk and the fresh milk (collected the next morning) are mixed in a copper cheese tank. When the temperature reaches 52°C, the cheese is wrapped with gauze for cutting, molding, pressing, and then, soaking in brine for 3 weeks (D’Incecco et al., 2020). Approximately 5 kg of water is evaporated during maturity. On the other hand, soft and semi-hard cheeses, such as feta cheese, are soaked in brine for a short period (Marino et al., 2017). For Kazak cheese making, the milk collection process is similar to that of Parmesan cheese, with few differences: In a goatskin bag, old yogurt may or may not be added as a starter, followed by fermentation into yogurt (Zheng et al., 2021). Next, this yogurt is boiled with stirring to evaporate water. The remaining curds are placed in a canvas bag, which is then hung outdoors to remove moisture further and solidify into fresh cheese. This fresh cheese is then cut into small pieces or made into a pie shape and finally placed on a bamboo board for 30–90 days of spontaneous ripening (Zheng et al., 2018b).

The acceptability of cheese to the final consumer largely depends on specific sensory characteristics, including flavor and aroma. The unique characteristics and special quality of cheese all depend on the various compounds and molecules that constitute it, including fatty acids, amines, ketones, free amino acids, alcohols, aldehydes, lactones, and sulfur compounds (Califano and Bevilacqua, 2000). Nevertheless, the presence of these molecules is associated with cheese-making factors, including climate, regional conditions, geographical position, technology used, the cheese-associated microbiota, and ripening conditions (Buffa et al., 2004; Soggiu et al., 2016; Pino et al., 2018). Four pathways, namely glycolysis, citrate utilization, proteolysis, and lipolysis, are involved in cheese flavor formation (Vlieg and Hugenholtz, 2007; Landaud et al., 2008). In addition to bacteria and mold in cheese, studies have indicated that *Geotrichum candidum* has expression characteristics associated with carbohydrate, lipid, and amino acid metabolism; whereas *Debaromyces hansenii* is involved in the metabolism of other amino acids (Ray et al., 2014; Monnet et al., 2016). Yeast also deaminates amino acids to the corresponding ketoacids and NH_3_, increasing the pH of the cheese (Beresford et al., 2001; El Sheikha and Montet, 2014). Flavor compound production relies on the milk-degrading enzymes of each fermenting strain and on the complementation of metabolic pathways between strains; the produced flavor compounds may enhance the cheese flavor quality and variety. Therefore, functional diversity – which is closely related to the complexity of the cheese microbiota – is crucial in the flavor compounds multiplicity produced during ripening (Irlinger and Mounier, 2009).

Traditionally fermented cheeses have complex microbial communities, multi-strain co-fermentation, complex metabolic mechanisms, and different flavor profiles. Therefore, microbes play a pivotal role in cheese flavor formation. The current review aims to provide a comprehensive overview of dynamics of the cheese microbiota in various cheese-making processes and technologies as well as understand the main biochemical pathways of cheese flavor formation, with a specific focus on the role of yeasts in cheese. Furthermore, this review provides important advances in understanding the effects of different cheese-making techniques and microbial diversity on cheese flavor and quality.

## General Process of Cheese Fermentation and Key Control Points

### Characteristics of Various Cheeses

More than 2,000 different cheese varieties exist in the world, of which more than 400 are more famous (Fox, 1993). Depending on the moisture content, cheese preservation and ripening methods can vary greatly (Table 1). Extra-hard cheeses, such as Parmesan and Romano, are produced from very hard curds. These cheeses are low in moisture, produced from partially skimmed milk, and matured slowly (over 1–2 years) by bacteria. For hard cheeses, such as cheddar and Kazak, the curd is acidified before salting and pressing, and their ripening period is 3–12 months, whereas for semi-hard cheese, this period is 2–3 months. Semi-soft cheeses (e.g., Limburger and blue cheese) are ripened using bacteria (*Brevibacterium*) and/or mold (*Penicillium*). During ripening, mold is primarily grown on the surface of some cheeses (e.g., Camembert) but under the surface of other such cheeses (e.g., blue cheese).

**Table 1:** Classification and main varieties of cheese.

Form facture	Moisture content/%	Mature microorganisms	Main cheese variety	Cheese flavor	Country of origin
Extra hard cheese	**25–35**	Bacterial	Parmesan	Fruit flavor and salt	Italy (Woo and Lindsay, 1984)
			Romano	Strong flavor	
Hard cheese	**35–45**	Bacterial: atmospheric hole	Emmanuel	Fruity aroma and taste stimulation	Switzerland (Anifantakis et al., 2009)
			Gruyere	Aromatic, rich, and smooth smell	
		Bacterial: no air holes	Cheddar	Walnut flavor	United Kingdom (Zerfiridis et al., 1984; Fox, 1993)
Semi-hard cheese	**45–50**	Bacterial: small air hole	Gouda	Caramel and creamy candy	Netherlands (Aishima and Nakai, 2010)
		Bacterial: no air holes	Edam	Sweet and nutty	
Semi-soft cheese	**42–55**	Bacterial	Brick	Spicy	German (Brackett et al., 1982)
			Limburg	Spicy	
		Mold	Roquefort	Strong salt aroma	France, Denmark (Woo et al., 1984)
			Blue	Strong spicy	
Soft cheese	**55–80**	Mold	Camembert	Mild	France (Kubíčková and Grosch, 1997)
		Not mature	Cottage	Mild	America (Goel et al., 1971)
			Cream	Slightly sour	

Bold values indicate the values of the header and moisture content.

Milk high in bacterial numbers may contain lactose-fermenting bacteria, which may interfere with milk acidification during cheese making. A cheese maker would not have strict control over the rate and extent of acidification during cheese making, which is one of the key components of successful cheese making. Pasteurization kills most bacteria capable of fermenting lactose, so it is sometimes necessary to use added starter for proper fermentation. Pasteurization kills most lactose fermenters and enables more stringent acidification control during the cheese-making process more stringent, in turn facilitating cheese quality control. Therefore, adding a starter is necessary for proper fermentation here. For some cheese varieties, especially cheddar, Parmesan, and aged Gouda, it is a common practice to add adjunct bacteria (mostly *Lactobacillus* species) in milk to produce unique, characteristic flavors. The added adjuncts may include bacterial species that inhibit undesirable bacteria in the cheese and possibly have probiotic effects. Mold-ripened cheeses (e.g., Camembert) and soft, surface-ripened cheeses (e.g., Limburger) do not offer sufficient protection against pathogen growth of because they do not meet the required criteria. These cheeses have high water activity and lose their acidity during ripening, both of which facilitate the growth of contaminants. The cheese uniformity, hardness, and shape are related to certain basic factors, crucial to ensure that cheese has the suitable ripening conditions and develops the ideal basic characteristics. Softness is associated with higher water content, higher fat content, and stronger protein breakdown ability. In contrast, hard cheeses have a firm shape. The variety of cheese that may be obtained is determined by raw milk characteristics, fresh curd preparation method, and microorganisms in the milk or curds (associated with the unique flavor and characteristics produced during cheese production and ripening). The types of microbes involved in cheese production or ripening are decided by the inoculated microbes, cheese production conditions, and environmental factors.

### Cheese Fermentation Processes

**Figure 1:**
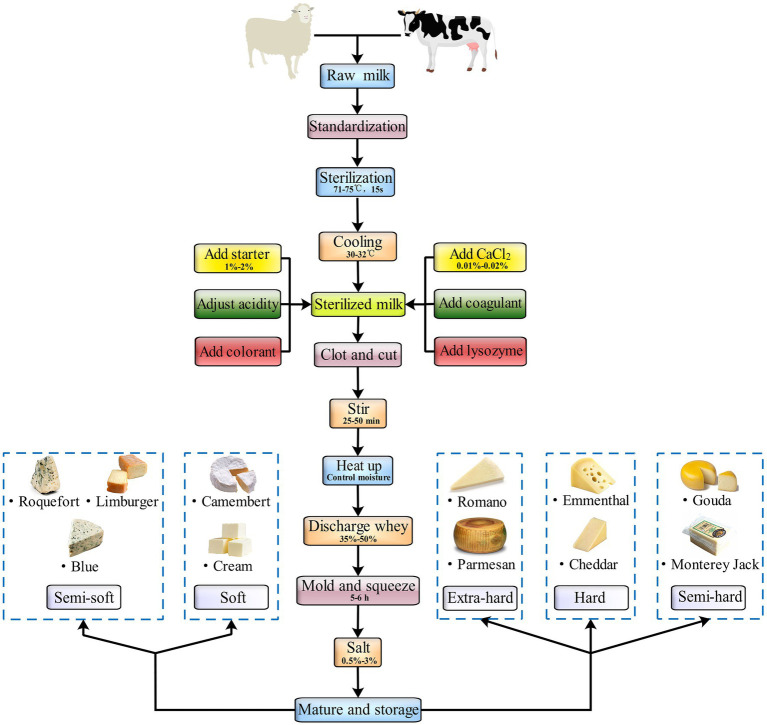
The general processing process of natural cheeses.

For centuries, the milk used to make many cheese varieties in the world has not pretreated in anyway before curdling. That is, raw milk, especially artisanal cheese, is conventionally used for making these cheeses (Kelly et al., 2008). Cheeses are traditionally manufactured by converting fluid milk to a semisolid mass by using a coagulating agent – such as rennet, acid, heat plus acid, or a combination of these. Cheeses can vary widely in their characteristics, including color, aroma, texture, flavor, and firmness, all of which are generally attributable to their production technology, milk source, moisture content, and aging length in addition to the presence of specific molds, yeast, and bacteria (Santiago-López et al., 2018). When milk is converted to cheese, some of the milk components are retained, whereas the others convert to unique components of the cheese. For instance, the microbial fermentation that milk undergoes during its transformation into cheese can modulate cheese composition directly *via* vitamin B synthesis (Reif et al., 1976). Microbial fermentation also indirectly regulates the composition of cheese by dissolving certain minerals and lactates lost in the whey after milk coagulation (Lucey and Fox, 1993). In addition, the composition of cheese changes according to the cheese-making process used (Mucchetti et al., 2008). Some of the major processes of natural cheese production are universal (Figure 1).

Cheese is generally produced from cow’s milk, but several cheeses, such as Roquefort, feta, and Manchego, are produced using the sheep or goat milk (Branciari et al., 2000). In general, raw milk has to be used for cheese production as soon as possible after it is expressed. However, delivering raw milk to a cheese factory in time can often be difficult in remote areas. Moreover, such factories may need to place the collected milk for 1 day before processing it. If the storage period ranges between 24 and 72 h, the bacterial numbers can increase to 10^6^ colony forming units/ml. The fat content in raw milk is determined by the fat content required in cheese, which in turn has a proportional relationship with the casein content of whole milk. Milk standardization can be done by adding cream, separating part of the fat, and adding skimmed milk or non–milk fat solids to achieve a uniform weight and reduce any deviation. To eliminate harmful and pathogenic bacteria, ensure uniform quality, and increase the stability of cheese quality, sterilized raw milk is used to produce most cheese varieties. The sterilization is performed at 63°C for 30 min or at 71–75°C for 15 s. Before cheese production, contaminating bacteria, mainly *Lactobacillus* spp., from the environment may ferment lactose; therefore, milk must be screened for acid and flavor compound–producing microbiota strains before the starter agents are added so as to maintain a stable acid production rate during clot formation and ensure cheese stability and quality. In addition to the starter, other agents can be added based on whether the cheese variety and production condition requires them; for instance, calcium chloride and colorants can be added to produce a curd with suitable hardness and consistent color. A clot can be only cut after it reaches appropriate hardness. Cutting allows for the conversion of large clots to smaller clots, thus speeding up the discharge of whey. It also increases the clot’s surface area, allowing for shrinkage *via* dehydration. As the firmness of the clot increases, its water-holding capacity decreases. Clot shrinkage and whey precipitation cause the clot to lose more water and become firmer. Generally, whey removal is performed to remove whey at a volume equal to 35–50% of the milk volume. The higher the temperatures during the whey discharge process, the higher is the clot moisture content. This is because clot particles deform quickly at high temperatures; as a result, the holes in the cheese particles close together rapidly, preventing water emission. Adding the proper content of salt during the cheese-making process can ensure proper acidity of the cheese, improve its texture properties and flavor, control the number of holes, adjust the moisture, and inhibit contaminating microbes (Upreti and Metzger, 2006). Regarding ripening and storage, fresh cheeses, such as cottage and cream cheese, do not need to be matured, whereas hard cheeses, such as cheddar and Swiss, do. Matured cheeses are generally made with a rennet curd. During ripening, unique flavors develop in a fresh clot through probiotic and enzymatic action, the intensity of which depends on the type of cheese (Moghaddas Kia et al., 2018). For ripening of extra-hard and hard cheeses such as Parmesan and cheddar, the clot is stored under conditions not conducive to the growth of surface microorganisms. This limits microbial and enzymatic activity. For instance, cheddar cheese is ripened in caves (Krishnan et al., 2019). For ripening all soft and some semi-soft cheeses – such as Limburger and Brie – the clots are stored under conditions that promote the growth of surface microorganisms. For instance, *Penicillium camemberti* is involved in the ripening of cheese such as Camembert and Brie, and *Brevibacterium linens* is involved in the ripening of spotted mature cheeses such as Limburg (Łopusiewicz et al., 2020). Interestingly, Blue cheeses such as Stilton and Roquefort are ripening in these two ways. In contrast, some traditional handmade cheeses, including Kazak and Plaisentif, do not have a specific standardized production process; they vary based on the traditional methods used by the cheese makers (Dalmasso et al., 2016; Zheng et al., 2021).

### Critical Control Points of Cheese

Extra-hard cheeses have very low water and fat contents. The key components of their production include low-fat milk, thermophilic LABs, high blanching temperature, long salt water–soaking period, and long-term slow ripening (Gobbetti and Di Cagno, 2017). For Parmesan, milk is collected in two batches: first, milk is left to stand overnight (to allow the fat to rise to the surface of the milk) and then churned to remove all the butter at the next morning. The remaining buttermilk is mixed with fresh milk. The milk is then fermented in a copper cheese tank. This process is similar to that Xinjiang Kazak cheese making, with only difference being that this cheese is fermented in a goatskin bag, not a copper tank (Zheng et al., 2018b). Next, after their formation, Parmesan clots are heated to about 52°C, and some part of the whey is scooped out as a starter for the next day’s cheese making; this part is essential for receiving a Parmigiano-Reggiano cheese certification (Neviani et al., 2013). Cheddar cheese, made from whole milk, contains 48% fat and 39% water and is fermented by the thermophilic *Streptococcus lactis*. It is produced at a low temperature, and it needs to reach a certain level of acidity before it is clotted and pressed. The clots are ground in the tearing or slicing mill, after which dry salt is added. Cheddar cheese is produced in way similar to Dutch cheese, with only difference being that the clot is repeatedly turned, stacked, and crushed. Since the high acidity of cheddar cheese during ripening inhibits the growth of butyric acid bacteria, nitrates are not added (Rilla et al., 2003).

The carbon dioxide produced by propionic acid bacteria (PAB) leads to formation of holes (also called “eyes”) in hard cheeses, such as Swiss and Emmental (Guggisberg et al., 2015). During the production of these cheeses, producing sufficiently elastic curds is critical. The ripening of Swiss cheese involves slow protein and lipid decomposition in the clot, which produces flavor substances and promotes the growth of PAB, which fermented lactates. The raw materials and starters used for Emmental are different from those used for other cheeses: the fresh milk used for making Emmental cheese is sourced from cows that are feed only grass and/or hay, no other feed or additives (Fröhlich-Wyder et al., 2002). Moreover, its starter culture consists of thermophilic *Lactobacillus* and *Propionibacterium*. However, few common limitations of using these raw materials include crack formation, few holes, pink spots, and insufficient flavor (Bertuzzi et al., 2018).

Semi-hard cheeses have a wide range of flavor profiles and structures because of the various LABs used and their effects. For Caerphilly and Lancashire cheeses, the growth of strains is promoted during the clot production stage: the low acidity (pH 5.0–5.2) of fresh cheese produces acidic clots that cause the cheese to have a crumbly texture (Jones et al., 2005; Tedeschi et al., 2013). In cheeses such as Edam and Gouda, a portion of the whey is discharged during mixing and replaced with water to limit acid production (Ibáñez et al., 2020). Therefore, the lactose content of the cheese decreases, its pH increase, and it develops firm but elastic structure. However, Gouda is made using pasteurized whole milk, whereas Edam is made using pasteurized or partially pasteurized buttermilk (Jo et al., 2018). Cleaning and removing whey are performed simultaneously during the manufacture process of Gouda – where some whey is removed and hot water is added to clean the curd. Finally, the moisture content in the cheese is controlled, and the final pH of it is also controlled by washing off the lactose to reduce the emission of lactic acid (Fox et al., 1990). In addition, Gouda is pressed further to remove whey and form a closed crust.

Limburger is called “smelly cheese” because of its strong aroma, mainly originating from the rind, rather than the cheese itself. Limburger is first stored for 2 weeks under higher temperature and humidity and then matured for 2–3 months under refrigerated conditions. During this time, it is soaked in brine several times to stimulate the growth of bacteria and the formation of a light brown crust and a unique taste. For storage, Limburger is wrapped in packaging made of breathable material such as aluminum foil or paper to ensure that the cheese remains ripe. Blue cheeses, including Roquefort and Danish blue cheese, are produced from high-acid, semi-soft curds, involving slow acid production over a long period of whey removal. The clots are not heated during processing, and the cheese is not mechanically pressed like pasta filata cheeses. The typical process of producing blue cheese includes puncturing the cheese for aeration to promote the growth of *Penicillium roqueforti*, which produces the typical blue lines. Camembert is a typical mature cheese with mold on the surface, which is soft and sticky. Traditional Camembert is made with fresh milk from local Normandy cows. After the cheese is basically shaped, dry salt is sprinkled on the surface, followed by inoculation with *Penicillium albicans*.

Mozzarella cheese is a semi-hard, fresh pasta filata family cheese. Its most unique processing technique includes hot stretching, which gives the cheese its distinctive texture. Mozzarella requires rapid acid production, but high acidity can also lead to low-quality cheese production. High-fat cheeses are sour curd cheeses. For these cheeses, whey is traditionally drained by hanging the clots in bags – similar to traditional Kazak cheese making (Zheng et al., 2018a). Feta cheese is made from sheep or goat milk, which is higher in short-chain fatty acids and thus produces a distinctive sour taste. However, the inherent lipase in this milk is destroy during the heat treatment process, and it is compensated for by adding lipase in the subsequent steps to ensure that the short-chain fatty acids generated *via* hydrolysis provide the unique sour taste.

### Development of Industrial Processing Technologies and Its Advantages

In contrast, the main forces that drive cheese technology are economics, equipment/engineering, consumer demands, and regulatory standards. In addition, production of consistent and high-quality cheese, while maintaining high-volume throughput is a key challenge in cheese manufacture (Panikuttira et al., 2018). Adoption of process analytical technology (PAT) for continuous monitoring and control of relevant processing parameters in dairy processing mini-mises the production of low-quality product and increases productivity and profitability (Tajammal Munir et al., 2015). Alternative processes for the reduction of bacterial load include the use of specially designed centrifuges or microfiltration. Hydrogen peroxide/catalase treatment of milk and Bactofugation (a high-speed centrifuge) could be used to remove bacteria and bacterial spores from milk (Legg et al., 2017). More recently, adjusting the protein content of the milk through the use of ultrafiltration technology was to achieve the desired final composition. Advantages include a more uniform starting material, profitable use of a lactose stream, and greater throughput of milk solids through the cheese vat. The choice of equipment for the vat stage of the cheese-making process depends on many external factors, including type of cheese to be made, downstream curd processing, flexibility, cost, and throughput, to name but a few.

In recent years, new biotechnologies to promote cheese maturation and improve flavor have been explored frequently, including the inoculation of additional cultures and exogenous enzymes, and the impact of temperature and high pressure on the quality of cheese (Khattab et al., 2019). Instrumental techniques and sensory panels can be expensive and require trained personnel warranting for more innovative, rapid-detection systems for ripening monitoring and evaluation of cheese quality such as infrared (IR) spectroscopy, electronic nose, and optical techniques. In order to increase consumer acceptance of processed cheese products, manufacturers are often seeking new ways to increase their functionality. The main cheese fortification methods are through the incorporation of probiotics and prebiotics, vitamin enhancement, and fortification of the PC with other macronutrients (Talbot-Walsh et al., 2018).

The development of dairy technology has allowed for the standardization of industrial production technology for some cheeses, whereas other cheeses are still prepared using with the traditional non-standardized methods. For example, ultrafiltration and concentration technologies are more suitable for feta production, but Kazak cheese is made using the traditional non-standardized manual processes. In addition, use of edible films and coatings in cheese preservation has opportunities and challenges (Costa et al., 2018).

## Research Progress on Cheese Flavor-Related Metabolic Mechanisms and Detectioin Technologies

### Cheese Flavor: Origin and Production

Cheese is a product of biochemical dynamics occurring during its production and ripening (McSweeney and Sousa, 2000). Each cheese has its unique flavor compound composition. Cheese flavor production mainly involves three main reactions: residual lactose, lactate, and citrate metabolism; proteolysis; and lipolysis (McSweeney, 2011). The enzymes involved in cheese production and ripening are mainly derived from the milk, the starter culture, rennet, and the secondary microbiota. Various changes in non–starter LAB (NSLAB) and secondary or adjunct cultures also occur depending on cheese types and processing methods. Food flavor substances are mainly volatile and nonvolatile. Volatile flavor substances include alcohols, acids, esters, aldehydes, and ketones – all of which are the source of food aroma (Marsili, 1985). In contrast, nonvolatile flavor substances mainly include organic acids, amino acids, reducing sugars, nucleotides, polypeptides, and other small molecules – all of which are the source of food taste (Delgado et al., 2010).

Cheese flavor substances include acids, alcohols, esters, ketones, and lactones – all of which are affected by raw milk quality and fermentation and/or ripening processes (Urbach, 1993; Santiago-López et al., 2018). In particular, ripening is the most important factor affecting cheese flavor. Cheese ripening process involves very complex biochemical reactions, which include primary and secondary metabolism. The basic flavor of cheese is determined by primary metabolism and mainly contains threes changes: carbohydrate decomposition, protein hydrolysis, and fat degradation. Secondary metabolism is responsible for the formation of flavor specific to a cheese variety. It mainly includes amino acid decarboxylation, transamination, deamination, de-sulfation, fatty acid beta-oxidation, and esterification (Marilley and Casey, 2004). Yeast can effectively produce many secondary metabolites crucial to the quality of cheese, including carbonyl compounds, sulfur compounds, fatty acid derivatives, phenolic compounds, and higher alcohols – which are directly related to cheese aromas (Dzialo et al., 2017).

### Residual Lactose, Lactate, and Citrate Metabolism

**Figure 2:**
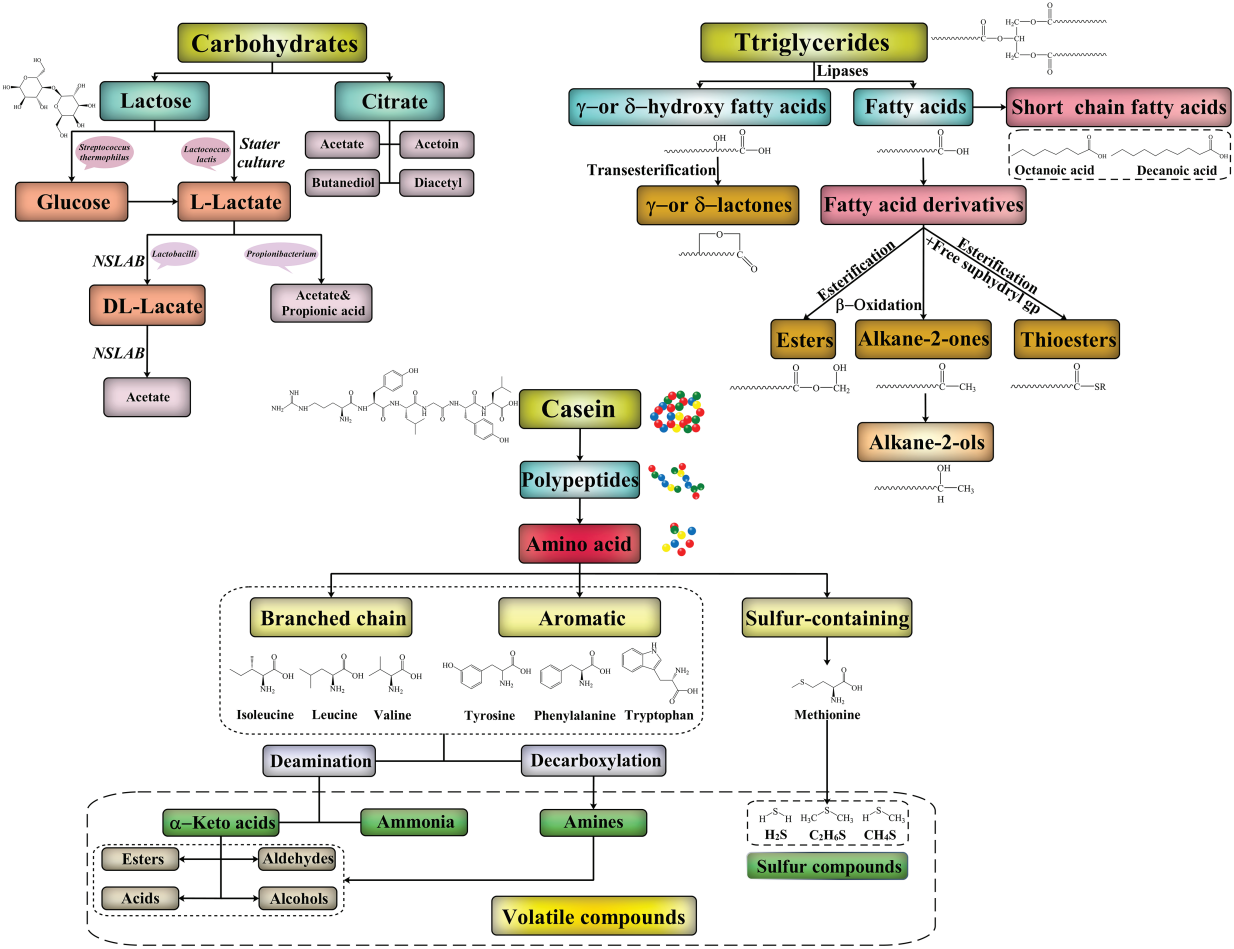
Biochemical pathways for production of flavor compounds in cheeses.

Lactose and citrate are the main carbohydrates in all mammalian milks, but the lactose content varies widely from mammal to mammal (range, 0–100 g/L; Lai et al., 2016; Figure 2). The principal products of lactose metabolism are L-lactate, DL-lactate, or a racemic mixture of both, which is essential to the flavor production in all cheeses. However, some bacteria including *Leuconostoc* spp. also produce other products such as ethanol (Vedamuthu, 1994). The nonstarter microbiota of cheddar, Dutch-type, and similar cheeses isomerize the L-lactate produced by the *Lactococcus lactis* starter to DL-lactate (McSweeney et al., 2017). However, a high DL-lactate concentration can affect the sensory quality of a cheese. Certain starter bacteria (e.g., *Streptococcus thermophilus*) growing with galactose-positive microorganisms cannot metabolize the galactose moiety of lactose, leading to galactose accumulation in the curds. Pyruvate – an intermediate of lactose metabolism – is the precursor for the production of several short-chain flavor compounds, including acetate, acetoin, diacetyl, ethanol, and acetaldehyde (Melchiorsen et al., 2002). In Swiss cheese, lactate is metabolized into propionates, acetates, carbon dioxide, and water by *Propionibacterium* spp., among which carbon dioxide is the cause of the characteristic “eyes” in the cheese (Duru et al., 2018). Acetate is an important flavor compound in many cheeses. In addition to being metabolized from lactose by LAB, acetate may also form *via* lactate and citrate metabolism (McSweeney and Sousa, 2000).

In mature cheeses such as Camembert and Brie, lactate in the surface layer is metabolized and decomposed into water and oxygen by the mold and yeast on the surface, causing their pH to increase (Lucey and Fox, 1993). This is similar to the changes occurring in Dutch and Swiss cheese, but not in cheddar. Lactose concentration in the cheese can drop as a result of washing or being replaced by whey; in this case, the remaining lactose in the clot is quickly metabolized with an increase in pH value. Consequently, cheeses with low-residue lactose have a fresh and mild flavor; whereas those with high-residue lactose may have a strong and pungent flavor because of low pH value.

In milk, citrate mainly exists in the form of ionized salts at concentrations of ≤1.8 g/L, most of which is lost in the whey during cheese making. This is because nearly 94% of the citrate is in the soluble phase of the milk (McSweeney et al., 2017). Citrate is not metabolized by *S. thermophilus* or thermophilic lactobacilli but by certain mesophilic lactobacilli in the NSLAB microbiota. Citrate is not metabolized by *S. thermophilus* or by thermophilic lactobacilli, but is metabolized by certain mesophilic lactobacilli in the NSLAB microbiota. A number of important flavors compounds, such as acetate, diacetyl, acetoin, butanediol, and carbon dioxide, are produced from citrate if some citrate-positive lactate (e.g., *L. lactis* and *Leuconostoc*) are promoted. Diacetyl is an important aroma compound converted to acetoin, 2,3-butanediol, and 2-butanone in some varieties of cheese, such as Dutch-type cheeses, quark, and cottage cheese (Dimos et al., 1996). Citrate metabolism is of particular importance in Dutch-type cheeses, where the CO_2_ produced is responsible for eye formation (McSweeney and Sousa, 2000). In addition, citrate provides the primary substrate for Cit^+^ starter cultures and NSLAB, and residual citrate metabolized by NSLAB may also lead to tissue laxity in some cheeses, such as cheddar (Fox et al., 2017).

### Lipolysis and Fatty Acid Metabolism

Lipolysis has an important effect on cheese flavor and texture (Voigt et al., 2012). Lipases in cheese are derived from milk, rennet, starter, auxiliary starter, nonstarter bacteria, and exogenous enzymes. Of all lipases, lipoprotein lipase is important in the development of flavor in raw milk cheeses, but it has little effect on the flavor of cheese made from pasteurized milk (Sert et al., 2014). The lipolytic enzymes present in LAB can hydrolyze a substrate to generate free fatty acid esters, triacylglycerides, diacylglycerides, and monoacylglycerides. LAB esterase is active for <C_18_-monoacylglycerides, with especially higher sensitivity to C_8_-monoacylglyceride, but has no effect on >C_6_-diacylglycerides (Holland et al., 2005). In addition, ethyl-butyric acid is generated by the transfer of butyl from triglycerides to ethanol *via* transferase in the cells of LAB (Tomita et al., 2018). The ability of PAB to decompose fat is 10–100 times that of LAB. In Swiss cheese, PAB play a key role in the conversion of lactate to acetate, the production of characteristic flavor *via* carbon dioxide, and the formation of free acids (Schwenninger et al., 2011).

The formation of typical flavor of cheese through lipolysis mainly reflects in the following: the ester bonds between triglycerides and fatty acids break under the action of lipase and monoglycerides, diglycerides, and free fatty acids are produced (Deeth and Touch, 2000; Figure 2). Fatty acids have an important influence on the flavor of cheese. During cheese fermentation and ripening, a series of fatty acids with medium and short carbon chains (C > 4) are formed after milk fat degradation. This leads to the formation of characteristic flavor substances in cheese, and these substances are important indicators that determine the maturity of the cheese. The oxidation of fatty acids, especially polyunsaturated fatty acids, can produce various unsaturated aldehydes with intense flavors. This can lead to an unpleasant smell associated with rancidity; it is observed in Gouda, cheddar, and Swiss cheese after they spoil (Forde and Fitzgerald, 2000). Nevertheless, lipolysis has a positive effect in most cheeses, including such as Parmesan, Emmental, blue cheeses, and Italian cheese (e.g., Romano; Thierry et al., 2017).

Fatty acids produced *via* lipolysis, especially free fatty acids such as acetic, octanoic, and decanoic acids, are cheese flavor substances. Moreover, the unique texture and hardness of cheeses result from continuous volatilization of water. Of the free fatty acids produced, acetic acid provides cheese with a sharp taste, but too much acetic acid can afford the cheese a vinegar-like odor. The flavors produced by fatty acids vary according to the differences of fatty acid types and contents in the many cheese types (Mallatou et al., 2003). Butyric acid is an important flavor compound in cheese such as Romano and cottage cheese, whereas the main characteristic flavor of Swiss cheese is propionic acid produced by PAB (Singh et al., 2003). Moreover, hexanoic acid is responsible for a sweaty, pungent, and rancid flavor (Carunchiawhetstine et al., 2003); octanoic acid imparts a goaty, waxy flavor; and decanoic acid affords a fatty, citrus odor (Gan et al., 2016).

Short-chain fatty acids provide strong characteristic flavors, some of which are precursors to flavor and are converted to other aromatic substances, including lactones and alcohols (Temizkan et al., 2015). The principal lactones in cheese are γ- and δ-lactones – which have five- and six-sided rings, respectively, and impart an intense aroma. The esters in cheese are produced by the esterification reaction between short-chain fatty acids and medium-long-chain fatty acids produced by the degradation of milk fat and primary and secondary alcohols produced by lactose fermentation or amino acid metabolism during fermentation (Fox et al., 2015). The esters in cheese are produced *via* the esterification reaction between short-chain fatty acids and medium–long-chain fatty acids produced during milk fat degradation. Moreover, primary and secondary alcohols are produced through lactose fermentation or amino acid metabolism during fermentation. The ester compounds in cheese play an important role in the formation of aromas that are sweet, fruity, and floral. However, an excessive amount of ethyl butyrate and ethyl caproate leads to an overpowering fruity flavor defect (Castada et al., 2019). In addition, thioesters (i.e., s-methyl thioacetate, thioethyl-2-methylpropanoate, and s-methyl thiobutyrate) produced through the reaction of free fatty acids with sulfhydryl groups impart a garlicky, sulfur-like, or eggy flavor (Iwasawa et al., 2014). Finally, β-oxidation and subsequent decarboxylation of free fatty acids in some cheeses (e.g., blue cheese) result in the formation of methyl ketones or alkan-2-ones, especially heptanone and nonanone (McSweeney et al., 2004).

### Proteolysis and Amino Acid Metabolism

Protein hydrolysis, a main biochemical reaction, is crucial to the formation of cheese flavor and has an important influence on the release and taste of cheese flavor during cheese ripening process (Gan et al., 2016). The peptides and free amino acids, decomposed from protein by protease in cheese, are precursors of many cheese flavor substances. Milk proteins mainly include caseins (α-, β-, and κ-casein), which are lost in whey because they are not degraded significantly. Casein hydrolysis is the most important biochemical pathway for flavor formation in hard and semi-hard cheeses. Proteinases and peptidases catalyze the cleavage of the polypeptide chains to produce free amino acids, some of which act as precursors to flavor compounds during cheese production and ripening (McSweeney, 2011).

Free amino acid content and metabolism in mature cheese play essential roles in cheese flavor development. Under the action of transaminase, deaminase, decarboxylase, and other enzymes, free amino acids in cheese are transformed into a series of volatile and nonvolatile flavor substances (e.g., ketones, aldehydes, acids, and alcohols) *via* deamination, amination, and decarboxylase (Fox and McSweeney, 1998; Figure 2). Methionine, leucine, and glutamic acid concentrations are typically considered the indicators of the degree of protein hydrolysis in cheese. The enzymatic removal of the amino terminus of amino acids leads to the formation of flavor and aromatic substances, such as 3-methyl-butanol, methionyl-propyl aldehyde, sulfides, and aromatic esters – which impart a malty, a baked potato-like, a pungent, and a flowery odor, respectively (Suzuki-Iwashima et al., 2020). Amino acid concentrations, especially those of glutamic acid and lysine, are significantly higher in Parmesan than in Gouda, cheddar, or Emmental. Starter LAB (SLAB), NSLB, and other bacterial strains can produce amino acid–metabolizing enzymes that specifically act on branched-chain, aromatic, or sulfur-containing amino acids (Forde and Fitzgerald, 2000).

Branched-chain amino acids – the precursors of aromatic compounds such as isobutyl ester, 3-methylbutanal, and 2-methylbutanal – are found in different cheeses (Curioni and Bosset, 2002). In addition, isoleucine, leucine, and valine can be decarboxylated into isobutyl ester, 2-methylacetaldehyde, and ketoisocaproate, all of which possess strong unpleasant aromas (McSweeney, 2011). Aromatic amino acid catabolism begins with a transamination step – where indole pyruvate, phenyl pyruvate, and p-hydroxy-phenyl pyruvate are produced from tryptophan, phenylalanine, and tyrosine, respectively. The conversion of tryptophan or phenylalanine in many hard and soft cheeses leads to the formation of benzaldehyde, which is characterized by a bitter almond flavor. Amino acids are also deaminated and decarboxylated to produce compounds such as α-keto acids, ammonia, and amines – which are further transformed into compounds such as alcohols, esters, and acids. Ammonia is also an important flavor substance in many cheeses, such as Camembert and Gruyère (Corsetti et al., 2001; Engel et al., 2001).

In cheese, methionine is converted to volatile sulfur compounds, such as methanethiol (which has a rancid flavor) as well as dimethyl sulfide and dimethyl trisulfide (which have a garlicky flavor); they represent the basic flavor substances in many cheese varieties (Smit et al., 2005). S-Compounds are the major contributors of the characteristic aroma of cheddar; they also contribute to the garlicky smell of a well-ripened Camembert (McSweeney and Sousa, 2000). In addition to producing flavor substance, proteolysis followed by oxidative decarboxylation of amino acids may produce low–molecular-weight biogenic amines (BA) – an excessive amount of which can cause adverse physiological reactions (Spano et al., 2010). Therefore, BA detection is a necessary part of cheese safety analysis.

### Progress in Cheese Flavor Detection Technology Research

The various cheeses have varied aromas and complex structures, the analysis of which mainly based on volatile component extraction. At present, the main extraction methods include distillation, solvent extraction, headspace capture method (HS), and solid-phase microextraction (SPME). Distillation is a relatively simple extraction technique; however, it is time and labor intensive (Wang et al., 2008). Solvent extraction method requires a relatively high amount of solvent. HS is relatively fast and easy to operate, but the concentration of volatiles present in the headspace can be low, which may limit the results. SPME is a fast and effective sample flavor material enrichment technology, which shows effective flavor substance sampling, separation, concentration, and enrichment. SPME is often used in conjunction with methods such as gas chromatography (GC)–mass spectrometry (MS) for the detection of volatile flavor constituents in food (Kataoka et al., 2000; Bertuzzi et al., 2018).

Gas chromatography–mass spectrometry, which plays a significant role in food flavor substance analysis, has been widely used in the detection of volatile and semi-volatile samples (El Sheikha and Hu, 2020). GC–MS technology has certain advantages in the application process. At present, HS plus SPME (HS-SPME) in combination with GC–MS has applied to analyze the flavor substances of cheese, white wine, rice wine, Pu’er tea, and beer (Plutowska and Wardencki, 2007). Delgado used SPME–GC–MS to analyze the volatile constituents of four different mature stages of the soft Spanish goat milk cheese (Delgado et al., 2010). A total of 46 volatile flavor compounds were detected, including 13 acids, nine esters, four ketones, seven alcohols, three aldehydes, seven aromatic compounds and others. Frenzel et al. (2015) used GC–MS to determine the volatile constituents of Italian Fiore Sardo PDO mature sheep cheese and detected carboxylic acid compounds (68%), while esters (14%), ketone (9%), and alcohol (8%) to be the main characteristic volatile compounds. Ceruti et al. (2016) used GC to separate volatile components from the ripening process of Reggianito cheese under different temperature–time combinations and isolated 41 volatile compounds including acid, ketones, aldehydes, esters, alcohols, and hydrocarbons.

More than 600 compounds have been identified as food volatile components thus far. Only a few of these compounds have a significant effect on the sensory flavor profile of the analyzed food. Only GC–MS can analyze for a wide range of volatile compounds. However, it cannot determine the flavor active ingredients that contribute the most to the flavor in food. Gas chromatography olfactometry (GC-O) is the most effective for detecting and identifying aroma component identification; the analytical methods that can be used with this technique mainly include the time–intensity method, Charm analysis, and aroma extraction dilution analysis (Zhu et al., 2015). In a time–intensity method, the key aromas in foods are identified by a sensory appraiser to describe the specific flavor of the volatile components they can smell. They then rank the aromas according to the intensity of the smell and the degree to which they contribute to the flavor. Research has shown that a combination of solvent extraction, SPME, and GC–O can be used to identify the key aroma compounds of blue-grain mature cheeses; the identified compounds include methyl mercaptan, 2(3)-methyl-butyric acid (cheese, pungent odor), 3-methylthiopropanal, 2,3-butanedione, dimethyl sulfide, butyric acid, 1-octene-3-ol (Z)-4-heptenal, phenylacetaldehyde, 2-ethyl-3,5-dimethylpyrazine, and acetic acid (Majcher et al., 2018).

## Yeast Diversity in Cheese and its Effects on Flavor

### Structural Diversity of Yeast Microbiota in Cheese

The traditional fermented foods, including Chinese liquor, cheese, vinegar, and bread, are enriched with various microorganisms in an open environment. Consequently, cooperative metabolism of multiple microbiotas underlies the fermentation involved in these foods (Wolfe et al., 2014). In addition, the microbial community structure and flavor of these foods are closely related. Therefore, a microbial community that inhabits in a cheese has a strong ecological adaptability and diversity. Microbial communities in traditional fermented foods also play an important role in food preservation and flavor formation (Wu et al., 2012). High-throughput metagenomics may reveal the diversity and succession of the microbial community on the surface of cheese – which is a food ecosystem with relatively simple microbiota. Moreover, metagenomics, macro-transcriptomics, and proteomics may be combined to greatly facilitate the mining of the metabolic functions of microbial communities in traditional fermented cheese (Almeida et al., 2014; Delcenserie et al., 2014; Gkatzionis et al., 2014). Combining pure cultivable and noncultivable methods (metagenomic techniques) to explore microbial communities has greatly increased out understanding of the microbiota on traditional fermented cheese (Aldrete-Tapia et al., 2014). However, few studies have focused on yeast strains in cheese, even though a diverse yeast microbiota inhabiting cheese has significant roles in cheese quality control.

The cheese ecosystem is a special habitat that supports the coexistence of yeast, bacteria, and filamentous fungi. The initial dominant yeasts are acid and salt tolerant; they can metabolize lactate produced by the SLAB and produce NH_3_ from amino acids. Yeast in cheese originates from not only milk but also the processing environment and storage process during the cheese fermentation process (Gonçalves Dos Santos et al., 2017). Yeasts that exist in the environment of raw milk and dairy products can easily settle on the surface of new cheese and form a complex biofilm along with other microorganisms (Fröhlich-Wyder et al., 2019). This phenomenon is usually seen in traditional mature cheeses. Numerous yeast species have been isolated from the surface of various cheeses; however, the roles they play in cheese ripening remains poorly understood. *Yarrowia lipolytica*, *D. hansenii*, *Kluyveromyces lactis*, and *Kluyveromyces marxianus* have been mainly isolated from French artisanal cheese surface and cores (Ceugniez et al., 2015). Around 137 different cheeses from 10 countries were collected and 24 cultivatable bacteria and fungi were found to be widely distributed in on cheese surface *via* metagenomic sequencing and strain isolation (Wolfe et al., 2014). Commercial yeast cultures, such as *G. candidum*, have been used in cheese production for many years. The use of yeast as an adjunct culture has also become popular in recent years. In the German Harzer and quark, the addition of highly active yeasts (*D. hansenii* and *Candida krusei*) can promote ripening (Fröhlich-Wyder et al., 2019). However, most of the yeasts found inside cheese are strictly anaerobic; these yeasts include *K. marxianus*, which metabolizes residual lactose.

A study on the diversity of 44 types of cheese fungi found that *D. hansenii* was the most abundant yeast and *Pe. roqueforti* was the most common mold (especially in blue cheese); moreover, most of the fungi were isolated from dairy products (Banjara et al., 2015). However, the potential safety issues associated with consumption of fungi such as *Aspergillus flavus* also require attention. Nineteen filamentous fungi and five yeast strains have identified to be part of traditional Turkish cheese fermentation; these especially include *Penicillum* spp. and *D. hansenii* (Budak et al., 2016). Moreover, *Lactobacillus*, *Lactococcus*, *Enterococcus*, and some yeasts have been found in PDO Ragusano cheese; these microbes play an important role in flavor formation (Carpino et al., 2017). In terms of fungi, Slovak cheese mainly contains *Hansenula debali*, *Y. lipolytica* and *G. candidum* (Chebeňová-Turcovská et al., 2011), whereas Livarot cheese mainly includes *Y. lipolytica*, *Candida* spp., *Candida intermedia*, and *Geotrichum* (as detected *via* fluorescence *in situ* hybridization; Mounier et al., 2009). *Pichia kudriavzevii* is the predominant yeast in Kazak cheese, followed by *K. marxianus* and *K. lactis* (Zheng et al., 2018a). A broad range of yeasts has been isolated from Camembert and Brie, with *D. hansenii* and *Y. lipolytica* being the most abundant isolated species (Viljoen et al., 2003). The main metabolic end-products of lactose and galactose fermentation by yeast isolated from water-buffalo Mozzarella cheese demonstrates great variability depending on the species (Suzzi et al., 2000). In general, *K. marxianus* and *K. lactis*, with their anamorphous species *D. hansenii* and *Saccharomyces cerevisiae*, are the most common yeasts in cheese, but their role in the cheese ripening process has not been fully evaluated.

### Effects of Yeast on Cheese Quality During Fermentation

Traditional fermented cheeses show complex microbial communities, multispecies cofermentation, complex metabolic mechanism, and varied flavors. The flavors in cheese are mainly produced *via* lactose and casein decomposition as well as lipid metabolism. In cheese ripening, most of the flavor substances are derived from protein hydrolysis and amino acid conversion (Engels et al., 1997). Moreover, the formation of flavor substances is inseparable from the metabolism of the microbiota and the conversion of substances during fermentation. A certain number of LAB is usually present in fresh milk and immature cheese, and some of the LABs have more highly active amino acid–converting enzymes; this increased the flavor diversity and richness in cheese (Centeno et al., 2002). Penicillium brevicompactum, Penicillium cavernicola, and Penicillium olsonii have a higher protease activity in handmade goat milk cheese, where Mucor produces more lipase. Moreover, *Y. lipolytica* has the best protease and lipase activity (Ozturkoglu-Budak et al., 2016). In addition to LAB and mold, various yeasts participate in protein hydrolysis, lipid and lactose degradation, and lactate and citrate assimilation during cheese ripening – all of which are important in cheese flavor formation (Fox and McSweeney, 1998; McSweeney, 2010; Padilla et al., 2014). Furthermore, *Metschnikowia reukaufii*, *Y. lipolytica*, and *Pi. kudriavzevii* affect the release of proteases, which was vital for the formation of free amino acids from proteins (Akpınar et al., 2011).

Traditional fermented cheeses have a stable core microbiota; however, the yeast species present in this microbiota needs to be further analyzed based on their metamorphic genome and metabolomics. The effects of microbial interactions, environment, and production processes on the microbial community of cheese have demonstrated that the microbes distributed in cheese surfaces are highly reproducible, making cheese an easy-to-handle, constructible microecosystem model. The focus of ongoing relevant research includes the following: (1) a method for effective control of the ripening stage of cheese for ensuring the flavor and quality of the finished cheese; (2) identification of the core microbiota including various yeast species involved and their interactions during cheese production and ripening; (3) exploration of the correlation between the aforementioned interactions or dynamic changes and the flavor changes during cheese production and ripening; and (4) a method to analyze the internal relationship between the ecological and functional characteristics of the cheese microbial community.

Yeasts play an important role in the manufacture of nearly all traditional ripened cheeses, especially some smear ripened cheeses such as Gruyère, Tilsit, and Reblochon (Rea et al., 2007). Some fermenting yeasts can grow in the interior of acid curd cheeses, such as the German Harzer; they produce ethanol and carbon dioxide in the early stage of manufacture (Fröhlich-Wyder et al., 2019). However, yeasts may also be the reason of some major defects in cheese, causing early blowing, an off flavor, brown discoloration, and other visible alterations (Jakobsen and Narvhus, 1996). Yeasts can perform deacidification at the surface of the cheese, causing a pH gradient to form between the surface and the center of the cheese, followed by outward lactate diffusion. When lactate depletes, yeasts break down amino acids to produce NH_3_, which diffuses inward and further increases the pH value (Gori et al., 2013; Monnet et al., 2015). The deacidification process contributes to the establishment of salt-tolerant, gram-positive, and catalase-positive bacterial communities with lower acid tolerance (Wolfe et al., 2014).

The development of yeast in cheese depends on many physicochemical conditions, such as low pH, mold content, high salt concentration, refrigerated ripening, and storage conditions (Viljoen et al., 2003). Yeasts that grow on the surface of cheese must be able to grow at low pH, low temperature, low water activity, and high salt concentrations (Monnet et al., 2015). For instance, using metagenomics, Filippis, et al. (2016) found changes in microbial communities and their functions during temperature-driven cheese ripening: increased microbial proteolysis, lipolysis, amino-acid and lipid catabolism–related gene expression, and cheese ripening rate. The distinct cheese flavors are produced according to the differences in fermentation conditions and starter cultures, which mainly contain bacterial and yeast starter cultures. Fungal starters, such as those containing *Pe. albicans* and *Pe. roqueforti*, can have proteolytic and lipolytic activities (Nielsen et al., 2005). Moreover, *Pe. albicans* produces white hyphae, whereas *Pe. roqueforti* can accelerates cheese ripening and produce a spicy flavor and a dark green color. In recent years, yeasts have also been used as assistant strains in starter cultures to produce cheese; for example, *Candida lipidotica* is used to produce blue cheese (Roostita and Fleet, 1996). As adjunct cultures, yeast species contribute to the development of flavor and texture during the production and ripening of certain cheese types. This is because their lipolytic and proteolytic activities can shorten the ripening time and thus enable economic cheese manufacture (Roostita and Fleet, 1996). In many cases, the most common yeasts in mold and bacterial surface–matured cheese include *Kluyveromyces*, *Debaryomyces*, and other species of *Saccharomyces*. Studies have found the presence of *Candida* spp., *Candida zeylanoides*, and dairy yeast in French Reblochon cheese; *D. hansenii* and *K. marxianus* yeasts in St. Nectaire cheese; and *Y. lipolytic*, *K. marxianus*, and *D. hansenii* in Tilsiter cheese (Corredor et al., 2000; Fröhlich-Wyder et al., 2019).

Most of the yeasts isolated from the surface of mature cheeses are salt tolerant, of which *D. hansenii* can tolerate high sodium chloride levels and utilize lactose, lactic, and citric acids for its proliferation (Gori et al., 2005; Breuer and Harms, 2006). Yeasts use residual lactate to deacidify the surface of cheese and produce vitamins and precursor substances, including niacin, riboflavin, and p-aminobenzoic acid, and thus promote *B. linens* growth on the cheese surface (Seiler and Busse, 1990). *Debaromyces hansenii* and *Y. lipolytica* are used to accelerate the ripening of cheddar cheese and enhance its flavor characteristics, whereas *Y. lipolytica* and *K. lactis* are employed for accelerating blue cheese ripening (Ferreira and Viljoen, 2003). *Kluyveromyces lactis* and *K. marxianus* are important components of the cheese microbiota because they can use the lactose left over from fermentation in the curd to produce carbon dioxide; this is beneficial for producing the open structure of cheeses such as Roquefort (Dias et al., 2014). *Kluyveromyces marxianus* can produce volatile aroma compounds through proteolytic and lipolytic activity in blue and bloomy rind cheeses and produce esters (fruity flavor compounds) and acetaldehyde from ethanol and carboxylic acids produced during lactose fermentation (Binetti et al., 2013). Research has shown that *Pi. kudriavzevii* N-X has the strongest extracellular proteolytic activity in skim milk agar and that it produces a range of aroma compounds (including ethanol, ethyl acetate, 3-methylbutanol, and acetic acid) in Kazak cheese (Zheng et al., 2018a). Research has also found that the texture of cheese with yeasts including *K. marxianus* and *Pi. kudriavzevii* added is relatively more brittle (Xiao et al., 2020). *Kluyveromyces marxianus* contributes to the formation of free amino acids and organic acids, especially glutamate and lactate. In addition, *K. marxianus* provides cheese with onion, oily, and floral aromas. Furthermore, *Pi. kudriavzevii* promotes a strong brandy, herbaceous and onion flavor. Both *K. lactis* and *Pichia fermentans* can ferment and constitute the typical yeast microbiota in feta (Rantsiou et al., 2008). Moreover, *Y. lipolytica* and *G. candidum* have important influences on its flavor during cheese production and ripening (Steensels and Verstrepen, 2014).

*Geotrichum candidum* strains can be found in inside and on the surface of cheese; they grow rapidly at the early stages of cheese ripening in Limburger, Tilsit, and Romano cheeses (Lessard et al., 2014; Banjara et al., 2015). However, they can metabolize galactose, but not lactose. In contrast, *D. hansenii* simultaneously metabolizes lactose and lactate, both of which are present in cheese at its early ripening stages (Mansour et al., 2008). Interestingly, *Y. lipolytica* is strictly aerobic, and it metabolizes lactate. *Geotrichum candidum* is the most active species involved in casein and fat degradation, which leads to an increased release of ammonia (Dugat-Bony et al., 2015). Recent studies have shown that *D. hansenii*, *G. candidum*, and *Y. lipolytica* may produce volatile compounds that contribute to cheese flavor, such as branched-chain aldehydes and alcohols (Sørensen et al., 2011; Padilla et al., 2014). *Yarrowia lipolytica* is found in foods with high protein or fat content because of its strong lipolytic and proteolytic activities (Nicaud, 2012). High amounts of volatile compounds, such as organic acids, sulfides, furans, and short-chain ketones, are produced by *Y. lipolytica* during cheese ripening (Sørensen et al., 2011). The coexistence of *Y. lipolytica* with *G. candidum* can have a negative effect on hypha formation. Moreover, *D. hansenii* and *Y. lipolytica* can dominate the yeast biota of smear ripened cheeses (Mounier et al., 2008; Atanassova et al., 2016; Ceugniez et al., 2017). In the presence of other yeasts, such as *Y. lipolytica* and *G. candidum*, the population of *D. hansenii* significantly decreases. However, *D. hansenii* may inhibit the growth of *K. lactis* and *G. candidum* (Lessard et al., 2012). Finally, the yeasts, bacteria, and mold in cheese may have a symbiotic effect that promotes the flavor development in cheese, but the underlying complex mechanism requires further analysis.

Traditional fermented cheeses are mostly fermented naturally using multiple microbial strains. Scientific problems such as unclear mechanism underlying flavor substance formation and unstable flavor quality have become a bottleneck for developing standardized cheese production processes – severely restricting the transformation of manual production to industrial processing. In recent years, studies have screened the functional microbial strains that improve the flavor of cheese. A study reported that the contents of ethanol, esterase, and glycol acyltransferase are the main factors limiting the synthesis of ethyl acetate in French Camembert (Hong et al., 2018). The results showed that adding *L. lactis* CCFM 12 with high esterase activity and ethanol acyltransferase activity significantly increased the contents of ethyl acetate and fruit aroma. Price et al. (2014) studied the effects of the addition of the functional yeasts *Y. lipolytica* and *K. lactis* on the aroma of blue cheese. These effects were studied by using model combining *Y. lipolytica*, *K. lactis*, and *Pe. roqueforti*, and the results indicated that the low inoculum of *K. lactis* was positively correlated with the cheese flavor, especially the formation of ketone compounds when a small inoculum of *Y. lipolytica* and *K. lactis* was used, the flavor of blue cheese was enhanced. Sørensen et al. (2011) inoculated a combination of *Y. lipolytica*, *S. cerevisiae*, and *D. hansenii* into the cheese fermentation process to improve the flavor quality of the cheese. Compared with the control group, *Y. lipolytica* mainly produced sulfides, furans, and short-chain ketones and *D. hansenii* significantly increased the content of branched-chain aldehydes and alcohols. Therefore, it is important to enhance the formation of flavor by strengthening these advantageous yeasts during the process of cheese ripening. During the process of cheese ripening, the proteolysis, lipolysis, and lactose degradation ability of yeast as well as their lactate and citrate assimilation play important roles – all of which are closely related to the formation of the cheese flavor (Fox and McSweeney, 1998). Therefore, how to maintain the flavor stability of handmade traditional cheese through the strengthening of functional yeasts will provide ideas for later research.

## Conclusion

With the advancement of science and technology, the use of various traditional methods may decrease. Thus, application and protection of microbial resources used in traditional fermented foods, such as cheese, is urgently needed. Therefore, evaluating the impact of dairy products on human health by using microbial resources in traditional fermented dairy products is essential. The microbial population inhabiting cheese has strong ecological adaptability; it also determines microbial community structural diversity and flavors of the various cheeses from different countries. The regional and climatic differences and diversification of processing technologies have induced considerable changes in the cheeses worldwide in terms of factors, such as appearance and flavor. The metabolic effects of yeast on cheese ripening and quality have long been underestimated, and the metabolic mechanisms of yeast have slowly been elucidated in the recent years. Therefore, studying the relationship of yeast community structure with the formation of yeast microbiota and flavor substances in the process of cheese fermentation is the key to producing cheese with the desired flavor and stable quality by using a cheese-specific standardized process. In summary, cheese has the potential to become a dairy product consumed on a large-scale in the future, and thus, it has very broad market prospects. The current review provided a theoretical basis for the succession and selection of functional yeast strains, and optimization of cheese processing technologies to improve flavor and quality of fermented cheese.

## Author Contributions

XZ wrote the main text of the manuscript. BW and XS supervised the research activities. All authors contributed to the article and approved the submitted version.

## Conflict of Interest

The authors declare that the research was conducted in the absence of any commercial or financial relationships that could be construed as a potential conflict of interest.

## Publisher’s Note

All claims expressed in this article are solely those of the authors and do not necessarily represent those of their affiliated organizations, or those of the publisher, the editors and the reviewers. Any product that may be evaluated in this article, or claim that may be made by its manufacturer, is not guaranteed or endorsed by the publisher.

## References

[ref1] Aishima T. Nakai S. (2010). Pattern recognition of GC profiles for classification of cheese variety. J. Food Sci. 52, 939–942. 10.1111/j.1365-2621.1987.tb14248.x

[ref2] Akpınar O. Uçar F. Yalçın H. T. (2011). Screening and regulation of alkaline extracellular protease and ribonuclease production of *Yarrowia lipolytica* strains isolated and identified from different cheeses in Turkey. Ann. Microbiol. 61, 907–915. 10.1007/s13213-011-0213-x

[ref3] Aldrete-Tapia A. Escobar-Ramírez M. C. Tamplin M. L. Hernández-Iturriaga M. (2014). High-throughput sequencing of microbial communities in poro cheese, an artisanal Mexican cheese. Food Microbiol. 44, 136–141. 10.1016/j.fm.2014.05.022, PMID: 25084655

[ref4] Almeida M. Hébert A. Abraham A. L. Rasmussen S. Monnet C. Pons N.. (2014). Construction of a dairy microbial genome catalog opens new perspectives for the metagenomic analysis of dairy fermented products. BMC Genomics 15:1101. 10.1186/1471-2164-15-1101, PMID: 25496341 PMC4320590

[ref5] Anifantakis E. M. Veinoglou B. C. Kandarakis J. G. (2009). Manufacture of Gruyère-type cheese with 50:50 rennet/swine pepsin. J. Dairy Res. 48, 513–518. 10.1017/s0022029900022019

[ref6] Atanassova M. R. Fernández-Otero C. Rodríguez-Alonso P. Fernández-No I. C. Garabal J. I. Centeno J. A. (2016). Characterization of yeasts isolated from artisanal short-ripened cows’ cheeses produced in Galicia (NW Spain). Food Microbiol. 53, 172–181. 10.1016/j.fm.2015.09.012, PMID: 26678145

[ref7] Azarnia S. Robert N. Lee B. (2006). Biotechnological methods to accelerate cheddar cheese ripening. Crit. Rev. Biotechnol. 26, 121–143. 10.1080/07388550600840525 16923531

[ref8] Banjara N. Suhr M. J. Hallen-Adams H. E. (2015). Diversity of yeast and mold species from a variety of cheese types. Curr. Microbiol. 70, 792–800. 10.1007/s00284-015-0790-1, PMID: 25694357

[ref9] Banks J. M. Brechany E. Y. Christie W. (1989). The production of low fat cheddar-type cheese. Int. J. Dairy Technol. 42, 6–9. 10.1111/j.1471-0307.1989.tb01699.x

[ref10] Beresford T. P. Fitzsimons N. A. Brennan N. L. Cogan T. M. (2001). Recent advances in cheese microbiology. Int. Dairy J. 11, 259–274. 10.1016/S0958-6946(01)00056-5

[ref11] Bertuzzi A. S. McSweeney P. L. H. Rea M. C. Kilcawley K. N. (2018). Detection of volatile compounds of cheese and their contribution to the flavor profile of surface-ripened cheese. Compr. Rev. Food Sci. Food Saf. 17, 371–390. 10.1111/1541-4337.12332, PMID: 33350078

[ref12] Binetti A. Carrasco M. Reinheimer J. Suárez V. (2013). Yeasts from autochthonal cheese starters: technological and functional properties. J. Appl. Microbiol. 115, 434–444. 10.1111/jam.12228 23600736

[ref13] Brackett R. E. Applebaum R. S. Wiseman D. W. Marth E. H. (1982). Fate of aflatoxin M1 in brick and limburger-like cheese. J. Food Prot. 45, 553–556. 10.4315/0362-028X-45.6.553, PMID: 30866219

[ref14] Branciari R. Nijman I. J. Plas M. E. Di Antonio E. Lenstra J. A. (2000). Species origin of milk in Italian mozzarella and Greek feta cheese. J. Food Prot. 63, 408–411. 10.4315/0362-028X-63.3.408, PMID: 10716574

[ref15] Breuer U. Harms H. (2006). *Debaryomyces hansenii*-an extremophilic yeast with biotechnological potential. Yeast 23, 415–437. 10.1002/yea.1374, PMID: 16652409

[ref16] Budak S. O. Figge M. J. Houbraken J. Vries R. P. D. (2016). The diversity and evolution of microbiota in traditional Turkish divle cave cheese during ripening. Int. Dairy J. 58, 50–53. 10.1016/j.idairyj.2015.09.011

[ref17] Buffa M. Guamis B. Saldo J. Trujillo A. J. (2004). Changes in organic acids during ripening of cheeses made from raw, pasteurized or high-pressure-treated goats’ milk. LWT 37, 247–253. 10.1016/j.lwt.2003.08.006

[ref18] Califano A. N. Bevilacqua A. E. (2000). Multivariate analysis of the organic acids content of gouda type cheese during ripening. J. Food Compos. Anal. 13, 949–960. 10.1006/jfca.2000.0930

[ref19] Carpino S. Randazzo C. L. Pino A. Russo N. Rapisarda T. Belvedere G.. (2017). Influence of PDO Ragusano cheese biofilm microbiota on flavour compounds formation. Food Microbiol. 61, 126–135. 10.1016/j.fm.2016.09.006, PMID: 27697162

[ref20] Carunchiawhetstine M. E. Karagul-Yuceer Y. Avsar Y. K. Drake M. A. (2003). Identification and quantification of character aroma components in fresh chevre-style goat cheese. J. Food Sci. 68, 2441–2447. 10.1111/j.1365-2621.2003.tb07043.x

[ref21] Castada H. Z. Hanas K. Barringer S. A. (2019). Swiss cheese flavor variability based on correlations of volatile flavor compounds, descriptive sensory attributes, and consumer preference. Foods 8:78. 10.3390/foods8020078 PMC6406939 30791411

[ref22] Centeno J. A. Tomillo F. J. Fernández-García E. Gaya P. Nuñez M. (2002). Effect of wild strains of *Lactococcus lactis* on the volatile profile and the sensory characteristics of ewes’ raw milk cheese. J. Dairy Sci. 85, 3164–3172. 10.3168/jds.S0022-0302(02)74404-4, PMID: 12512589

[ref23] Ceruti R. J. Zorrilla S. E. Sihufe G. A. (2016). Volatile profile evolution of Reggianito cheese during ripening under different temperature–time combinations. Eur. Food Res. Technol. 242, 1–10. 10.1007/s00217-016-2640-1

[ref24] Ceugniez A. Drider D. Jacques P. Coucheney F. (2015). Yeast diversity in a traditional French cheese “Tomme d’orchies” reveals infrequent and frequent species with associated benefits. Food Microbiol. 52, 177–184. 10.1016/j.fm.2015.08.001, PMID: 26338133

[ref25] Ceugniez A. Taminiau B. Coucheney F. Jacques P. Delcenserie V. Daube G.. (2017). Fungal diversity of “Tomme d’Orchies” cheese during the ripening process as revealed by a metagenomic study. Int. J. Food Microbiol. 258, 89–93. 10.1016/j.ijfoodmicro.2017.07.015, PMID: 28806689

[ref26] Chebeňová-Turcovská V. Ženišová K. Kuchta T. Pangallo D. Brežná B. (2011). Culture-independent detection of microorganisms in traditional Slovakian bryndza cheese. Int. J. Food Microbiol. 150, 73–78. 10.1016/j.ijfoodmicro.2011.07.020, PMID: 21849217

[ref27] Corredor M. Davila A.-M. Gaillardin C. Casaregola S. (2000). DNA probes specific for the yeast species *Debaryomyces hansenii*: useful tools for rapid identification. FEMS Microbiol. Lett. 193, 171–177. 10.1111/j.1574-6968.2000.tb09420.x, PMID: 11094297

[ref28] Corsetti A. Rossi J. Gobbetti M. (2001). Interactions between yeasts and bacteria in the smear surface-ripened cheeses. Int. J. Food Microbiol. 69, 1–10. 10.1016/S0168-1605(01)00567-0, PMID: 11589547

[ref29] Costa M. J. Maciel L. C. Teixeira J. A. Vicente A. A. Cerqueira M. A. (2018). Use of edible films and coatings in cheese preservation: opportunities and challenges. Food Res. Int. 107, 84–92. 10.1016/j.foodres.2018.02.013, PMID: 29580546

[ref30] Curioni P. M. G. Bosset J. O. (2002). Key odorants in various cheese types as determined by gas chromatography-olfactometry. Int. Dairy J. 12, 959–984. 10.1016/S0958-6946(02)00124-3

[ref31] Dalmasso A. Soto Del Rio M. L. D. Civera T. Pattono D. Cardazzo B. Bottero M. T. (2016). Characterization of microbiota in Plaisentif cheese by high-throughput sequencing. LWT 69, 490–496. 10.1016/j.lwt.2016.02.004

[ref32] Deeth H. C. Touch V. (2000). Methods for detecting lipase activity in milk and milk products. Aust. J. Dairy Technol. 55:153.

[ref33] Delcenserie V. Taminiau B. Delhalle L. Nezer C. Doyen P. Crevecoeur S.. (2014). Microbiota characterization of a Belgian protected designation of origin cheese, Herve cheese, using metagenomic analysis. J. Dairy Sci. 97, 6046–6056. 10.3168/jds.2014-8225, PMID: 25064656

[ref34] Delgado F. J. González-Crespo J. Cava R. García-Parra J. Ramírez R. (2010). Characterisation by SPME–GC–MS of the volatile profile of a Spanish soft cheese P.D.O. Torta del Casar during ripening. Food Chem. 118, 182–189. 10.1016/j.foodchem.2009.04.081

[ref35] Dias O. Pereira R. Gombert A. K. Ferreira E. C. Rocha I. (2014). iOD907, the first genome-scale metabolic model for the milk yeast *Kluyveromyces lactis*. Biotechnol. J. 9, 776–790. 10.1002/biot.201300242, PMID: 24777859

[ref36] Dimos A. Urbacha G. E. Miller A. J. (1996). Changes in flavour and volatiles of full-fat and reducedfat cheddar cheeses during maturation. Int. Dairy J. 6, 981–995. 10.1016/S0958-6946(97)84214-8

[ref37] D’Incecco P. Limbo S. Hogenboom J. Rosi V. Gobbi S. Pellegrino L. (2020). Impact of extending hard-cheese ripening: a multiparameter characterization of parmigiano reggiano cheese ripened up to 50 months. Foods 9:268. 10.3390/foods9030268, PMID: 32131400 PMC7143483

[ref39] Dugat-Bony E. Straub C. Teissandier A. Onésime D. Loux V. Monnet C.. (2015). Overview of a surface-ripened cheese community functioning by meta-omics analyses. PLoS One 10:e0124360. 10.1371/journal.pone.0124360, PMID: 25867897 PMC4395090

[ref40] Duru I. C. Laine P. Andreevskaya M. Paulin L. Kananen S. Tynkkynen S.. (2018). Metagenomic and metatranscriptomic analysis of the microbial community in swiss-type Maasdam cheese during ripening. Int. J. Food Microbiol. 281, 10–22. 10.1016/j.ijfoodmicro.2018.05.017, PMID: 29803134

[ref41] Dzialo M. C. Park R. Steensels J. Lievens B. Verstrepen K. J. (2017). Physiology, ecology and industrial applications of aroma formation in yeast. FEMS Microbiol. Rev. 41, S95–S128. 10.1093/femsre/fux031, PMID: 28830094 PMC5916228

[ref42] El Sheikha A. F. (2018). “Revolution in fermented food: from artisan household technology to era of biotechnology,” in Molecular Techniques in Food Biology: Safety, Biotechnology, Authenticity & Traceability. eds. El Sheikha A. F. Levin R. Xu J. P. (Chichester, UK: John Wiley & Sons Ltd.), 241–260.

[ref43] El Sheikha A. F. Hu D. M. (2020). Molecular techniques reveal more secrets of fermented foods. Crit. Rev. Food Sci. Nutr. 60, 11–32. 10.1080/10408398.2018.1506906, PMID: 30296166

[ref44] El Sheikha A. F. Montet D. (2014). “African fermented foods: historical roots and real benefits,” in Microorganisms and Fermentation of Traditional Foods. eds. Ray R. C. Montet D. (Boca Raton, Florida, USA: Science Publishers Inc. and CRC Press), 248–282.

[ref45] Engel E. Septier C. Leconte N. Salles C. Le Quere J.-L. (2001). Determination of taste-active compounds of a bitter camembert cheese by omission tests. J. Dairy Res. 68, 675–688. 10.1017/S0022029901005209, PMID: 11928963

[ref46] Engels W. J. M. Dekker R. de Jong C. Neeter R. Visser S. (1997). A comparative study of volatile compounds in the water-soluble fraction of various types of ripened cheese. Int. Dairy J. 7, 255–263. 10.1016/s0958-6946(97)00003-4

[ref47] Ferreira A. D. Viljoen B. C. (2003). Yeasts as adjunct starters in matured Cheddar cheese. Int. J. Food Microbiol. 86, 131–140. 10.1016/S0168-1605(03)00252-6, PMID: 12892928

[ref48] Filippis F. D. Genovese A. Ferranti P. Gilbert J. A. Ercolini D. (2016). Metatranscriptomics reveals temperature-driven functional changes in microbiome impacting cheese maturation rate. Sci. Rep. 6:21871. 10.1038/srep21871, PMID: 26911915 PMC4766472

[ref49] Forde A. Fitzgerald G. F. (2000). Biotechnological approaches to the understanding and improvement of mature cheese flavour. Curr. Opin. Biotechnol. 11, 484–489. 10.1016/S0958-1669(00)00130-0, PMID: 11024368

[ref50] Fox P. F. (ed.) (1993). “Cheese: an overview,” in Cheese: Chemistry, Physics and Microbiology. Boston: Springer, 1–36.

[ref51] Fox P. F. Guinee T. P. Cogan T. M. McSweeney P. L. H. (eds.) (2017). “Starter cultures,” in Fundamentals of Cheese Science. Boston: Springer, 121–183.

[ref52] Fox P. F. Lucey J. A. Cogan T. M. (1990). Glycolysis and related reactions during cheese manufacture and ripening. Crit. Rev. Food Sci. Nutr. 29, 237–253. 10.1080/10408399009527526, PMID: 2257078

[ref53] Fox P. F. McSweeney P. L. H. (1998). Dairy chemistry and biochemistry. Mol. Asp. Med. 24, 3–9.

[ref54] Fox P. F. Uniacke-Lowe T. McSweeney P. O’Mahony J. A. (2015). “Chemistry and biochemistry of cheese,” in Dairy Chemistry and Biochemistry. ed. Fox P. F. (New York: Springer), 499–546.

[ref55] Frenzel M. Zerge K. Clawin-Rädecker I. Lorenzen P. C. (2015). Comparison of the galacto-oligosaccharide forming activity of different β-galactosidases. LWT Food Sci. Technol. 60, 1068–1071. 10.1016/j.lwt.2014.10.064

[ref56] Fröhlich-Wyder M.-T. Arias Roth E. Jakob E. (2019). Cheese yeasts. Yeast 36, 129–141. 10.1002/yea.3368, PMID: 30512214

[ref57] Fröhlich-Wyder M.-T. Bachmann H. P. Casey M. G. (2002). Interaction between propionibacteria and starter/non-starter lactic acid bacteria in swiss-type cheeses. Lait 82, 1–15. 10.1051/lait:2001001

[ref58] Gan H. H. Yan B. Linforth R. S. T. Fisk I. D. (2016). Development and validation of an APCI-MS/GC–MS approach for the classification and prediction of cheddar cheese maturity. Food Chem. 190, 442–447. 10.1016/j.foodchem.2015.05.096, PMID: 26212994 PMC4577651

[ref59] Gkatzionis K. Yunita D. Linforth R. S. T. Dickinson M. Dodd C. E. R. (2014). Diversity and activities of yeasts from different parts of a stilton cheese. Int. J. Food Microbiol. 177, 109–116. 10.1016/j.ijfoodmicro.2014.02.016, PMID: 24631634

[ref60] Gobbetti M. Di Cagno R. (2017). “Chapter 32: Extra-hard varieties,” in Cheese. 4th *Edn*. eds. McSweeney P. L. H. Fox P. F. Cotter P. D. Everett D. W. (San Diego: Academic Press), 809–828.

[ref61] Goel M. C. Kulshrestha D. C. Marth E. H. Francis D. W. Bradshaw J. G. Read R. B. (1971). Fate on coliforms in yogurt, buttermilk, sour cream, and cottage cheese during refrigerated storage. J. Milk Food Technol. 34, 54–58. 10.4315/0022-2747-34.1.54

[ref38] Gonçalves Dos Santos M. T. P. Benito M. J. Córdoba M. D. G. Alvarenga N. Ruiz-Moyano Seco de Herrera S. (2017). Yeast community in traditional Portuguese Serpa cheese by culture-dependent and -independent DNA approaches. Int. J. Food Microbiol. 262, 63–70. 10.1016/j.ijfoodmicro.2017.09.013 28964999

[ref62] Gori K. Mortensen H. D. Arneborg N. Jespersen L. (2005). Expression of the GPD1 and GPP2 orthologues and glycerol retention during growth of *Debaryomyces hansenii* at high NaCl concentrations. Yeast 22, 1213–1222. 10.1002/yea.1306, PMID: 16278930

[ref63] Gori K. Ryssel M. Arneborg N. Jespersen L. (2013). Isolation and identification of the microbiota of Danish farmhouse and industrially produced surface-ripened cheeses. Microb. Ecol. 65, 602–615. 10.1007/s00248-012-0138-3, PMID: 23224222 PMC3621994

[ref64] Guggisberg D. Schuetz P. Winkler H. Amrein R. Jakob E. Fröhlich-Wyder M.-T.. (2015). Mechanism and control of the eye formation in cheese. Int. Dairy J. 47, 118–127. 10.1016/j.idairyj.2015.03.001

[ref65] Holland R. Liu S. Q. Crow V. L. Delabre M. L. Lubbers M. Bennett M.. (2005). Esterases of lactic acid bacteria and cheese flavour: milk fat hydrolysis, alcoholysis and esterification. Int. Dairy J. 15, 711–718. 10.1016/j.idairyj.2004.09.012

[ref66] Hong Q. Liu X. M. Hang F. Zhao J. X. Zhang H. Chen W. (2018). Screening of adjunct cultures and their application in ester formation in camembert-type cheese. Food Microbiol. 70, 33–41. 10.1016/j.fm.2017.08.009, PMID: 29173637

[ref67] Ibáñez R. A. Govindasamy-Lucey S. Jaeggi J. J. Johnson M. E. McSweeney P. L. H. Lucey J. A. (2020). Low-and reduced-fat milled curd, direct-salted gouda cheese: comparison of lactose standardization of cheesemilk and whey dilution techniques. J. Dairy Sci. 103, 1175–1192. 10.3168/jds.2019-17292, PMID: 31864749

[ref68] Irlinger F. Mounier J. (2009). Microbial interactions in cheese: implications for cheese quality and safety. Curr. Opin. Biotechnol. 20, 142–148. 10.1016/j.copbio.2009.02.016, PMID: 19342218

[ref69] Iwasawa A. Suzuki-Iwashima A. Iida F. Shiota M. (2014). Effects of flavor and texture on the desirability of Cheddar cheese during ripening. Food Sci. Technol. Res. 20, 23–29. 10.3136/fstr.20.23 26551333

[ref70] Jakobsen M. Narvhus J. (1996). Yeasts and their possible beneficial and negative effects on the quality of dairy products. Int. Dairy J. 6, 755–768. 10.1016/0958-6946(95)00071-2

[ref71] Jo Y. Benoist D. M. Ameerally A. Drake M. A. (2018). Sensory and chemical properties of gouda cheese. J. Dairy Sci. 101, 1967–1989. 10.3168/jds.2017-13637, PMID: 29274971

[ref72] Jones E. L. Shingfield K. J. Kohen C. Jones A. K. Lupoli B. Grandison A. S.. (2005). Chemical, physical, and sensory properties of dairy products enriched with conjugated linoleic acid. J. Dairy Sci. 88, 2923–2937. 10.3168/jds.S0022-0302(05)72973-8, PMID: 16027207

[ref73] Kataoka H. Lord H. L. Pawliszyn J. (2000). Applications of solid-phase microextraction in food analysis. J. Chromatogr. A 880, 35–62. 10.1016/S0021-9673(00)00309-5, PMID: 10890509

[ref74] Kelly A. L. Huppertz T. Sheehan J. J. (2008). Pre-treatment of cheese milk: principles and developments. Dairy Sci. Technol. 88, 549–572. 10.1051/dst:2008017

[ref75] Khattab A. R. Guirguis H. A. Tawfik S. M. Farag M. A. (2019). Cheese ripening: a review on modern technologies towards flavor enhancement, process acceleration and improved quality assessment. Trends Food Sci. Technol. 88, 343–360. 10.1016/j.tifs.2019.03.009

[ref76] Kourkoutas Y. Bosnea L. Taboukos S. Baras C. Lambrou D. Kanellaki M. (2006). Probiotic cheese production using *lactobacillus casei* cells immobilized on fruit pieces. J. Dairy Sci. 89, 1439–1451. 10.3168/jds.S0022-0302(06)72212-3, PMID: 16606715

[ref77] Krishnan K. Campbell Y. L. To K. V. Lima G. Byron M. D. Zhang X.. (2019). Effects of temperature, relative humidity, and protective netting on Tyrophagus putrescentiae (schrank) (sarcoptiformes: Acaridae) infestation, fungal growth, and product quality of cave-aged Cheddar cheese. J. Stored Prod. Res. 83, 44–53. 10.1016/j.jspr.2019.05.014

[ref78] Kubíčková J. Grosch W. (1997). Evaluation of potent odorants of camembert cheese by dilution and concentration techniques. Int. Dairy J. 7, 65–70. 10.1016/S0958-6946(96)00044-1

[ref79] Lai F. N. Zhai H. L. Cheng M. Ma J. Y. Cheng S. F. Ge W.. (2016). Whole-genome scanning for the litter size trait associated genes and SNPs under selection in dairy goat (*Capra hircus*). Sci. Rep. 6:38096. 10.1038/srep38096, PMID: 27905513 PMC5131482

[ref80] Landaud S. Helinck S. Bonnarme P. (2008). Formation of volatile sulfur compounds and metabolism of methionine and other sulfur compounds in fermented food. Appl. Microbiol. Biotechnol. 77, 1191–1205. 10.1007/s00253-007-1288-y 18064452

[ref81] Lawrence R. C. Gilles J. Creamer L. K. Crow V. L. Heap H. A. Honoré C. G.. (2004). “Cheddar cheese and related dry-salted cheese varieties,” in Cheese: Chemistry, Physics and Microbiology. eds. Fox P. F. McSweeney P. L. H. Cogan T. M. Guinee T. P. (Boston, MA: Springer), 71–102.

[ref82] Legg A. K. Carr A. J. Bennett R. J. Johnston K. A. (2017). “Chapter 26: General aspects of cheese technology,” in Cheese. 4th *Edn*. eds. McSweeney P. L. H. Fox P. F. Cotter P. D. Everett D. W. (San Diego: Academic Press), 643–675.

[ref83] Lessard M.-H. Bélanger G. St-Gelais D. Labrie S. (2012). The composition of camembert cheese-ripening cultures modulates both mycelial growth and appearance. Appl. Environ. Microbiol. 78:1813. 10.1128/AEM.06645-11, PMID: 22247164 PMC3298135

[ref84] Lessard M.-H. Viel C. Boyle B. St-Gelais D. Labrie S. (2014). Metatranscriptome analysis of fungal strains Penicillium camemberti and Geotrichum candidumreveal cheese matrix breakdown and potential development of sensory properties of ripened camembert-type cheese. BMC Genomics 15:235. 10.1186/1471-2164-15-235, PMID: 24670012 PMC3986886

[ref85] Łopusiewicz Ł. Drozłowska E. Tarnowiecka-Kuca A. Bartkowiak A. Mazurkiewicz-Zapałowicz K. Salachna P. (2020). Biotransformation of flaxseed oil cake into bioactive camembert-analogue using lactic acid bacteria, *Penicillium camemberti* and *Geotrichum candidum*. Microorganisms 8:1266. 10.3390/microorganisms8091266, PMID: 32825460 PMC7565573

[ref86] Lucey J. A. Fox P. F. (1993). Importance of calcium and phosphate in cheese manufacture: a review. J. Dairy Sci. 76, 1714–1724. 10.3168/jds.S0022-0302(93)77504-9

[ref87] Majcher M. A. Myszka K. Gracka A. Grygier A. Jeleń H. H. (2018). Key odorants of Lazur, a polish mold-ripened cheese. J. Agric. Food Chem. 66, 2443–2448. 10.1021/acs.jafc.6b04911, PMID: 28145120

[ref88] Mallatou H. Pappa E. Massouras T. (2003). Changes in free fatty acids during ripening of Teleme cheese made with ewes’, goats’, cows’ or a mixture of ewes’ and goats’ milk. Int. Dairy J. 13, 211–219. 10.1016/S0958-6946(02)00153-X

[ref89] Mansour S. Beckerich J. M. Bonnarme P. (2008). Lactate and amino acid catabolism in the cheese-ripening yeast *Yarrowia lipolytica*. Appl. Environ. Microbiol. 74:6505. 10.1128/AEM.01519-08, PMID: 18776032 PMC2576680

[ref90] Marilley L. Casey M. G. (2004). Flavours of cheese products: metabolic pathways, analytical tools and identification of producing strains. Int. J. Food Microbiol. 90, 139–159. 10.1016/S0168-1605(03)00304-0, PMID: 14698096

[ref91] Marino M. Innocente N. Maifreni M. Mounier J. Cobo-Díaz J. F. Coton E.. (2017). Diversity within Italian cheesemaking brine-associated bacterial communities evidenced by massive parallel 16S rRNA gene tag sequencing. Front. Microbiol. 8:2119. 10.3389/fmicb.2017.02119, PMID: 29163411 PMC5675859

[ref92] Marsili R. (1985). Monitoring chemical changes in Cheddar cheese during aging by high performance liquid chromatography and gas chromatography techniques. J. Dairy Sci. 68, 3155–3161. 10.3168/jds.S0022-0302(85)81221-2

[ref93] McSweeney P. L. H. (2010). Biochemistry of cheese ripening. Int. J. Dairy Technol. 57, 127–144. 10.1111/j.1471-0307.2004.00147.x

[ref94] McSweeney P. L. H. (2011). “Encyclopedia of dairy sciences (second edition),” in Cheese Biochemistry of Cheese Ripening. ed. Fuquay J. W. (San Diego: Academic Press), 667–674.

[ref95] McSweeney P. L. H. Fox P. F. Ciocia F. (2017). “Chapter 16: Metabolism of residual lactose and of lactate and citrate,” in Cheese. 4th *Edn*. eds. McSweeney P. L. H. Fox P. F. Cotter P. D. Everett D. W. (San Diego: Academic Press), 411–421.

[ref96] McSweeney P. L. H. Ottogalli G. Fox P. F. (2004). “Cheese: chemistry, physics and microbiology,” in Diversity of Cheese Varieties: An Overview. eds. Fox P. F. McSweeney P. L. H. Cogan T. M. Guinee T. P. (San Diego: Academic Press), 1–23.

[ref97] McSweeney P. L. H. Sousa M. J. (2000). Biochemical pathways for the production of flavour compounds in cheeses during ripening: a review. Lait 80, 293–324. 10.1051/lait:2000127

[ref98] Melchiorsen R. C. Jokumsen V. K. Villadsen J. Israelsen H. Arnau J. (2002). The level of pyruvate-formate lyase controls the shift from homolactic to mixed-acid product formation in *Lactococcus lactis*. Appl. Microbiol. Biotechnol. 58, 338–344. 10.1007/s00253-001-0892-5, PMID: 11935185

[ref99] Moghaddas Kia E. Alizadeh M. Esmaiili M. (2018). Development and characterization of probiotic UF feta cheese containing *lactobacillus paracasei* microencapsulated by enzyme based gelation method. J. Food Sci. Technol. 55, 3657–3664. 10.1007/s13197-018-3294-8, PMID: 30150825 PMC6098783

[ref100] Monnet C. Dugat-Bony E. Swennen D. Beckerich J. M. Irlinger F. Fraud S.. (2016). Investigation of the activity of the microorganisms in a reblochon-style cheese by metatranscriptomic analysis. Front. Microbiol. 7:536. 10.3389/fmicb.2016.00536, PMID: 27148224 PMC4837152

[ref101] Monnet C. Landaud S. Bonnarme P. Swennen D. (2015). Growth and adaptation of microorganisms on the cheese surface. FEMS Microbiol. Lett. 362, 1–9. 10.1093/femsle/fnu025, PMID: 25790503

[ref102] Monti L. Negri S. Meucci A. Stroppa A. Galli A. Contarini G. (2017). Lactose, galactose and glucose determination in naturally “lactose free” hard cheese: HPAEC-PAD method validation. Food Chem. 220, 18–24. 10.1016/j.foodchem.2016.09.185, PMID: 27855887

[ref103] Mounier J. Monnet C. Jacques N. Antoinette A. Irlinger F. (2009). Assessment of the microbial diversity at the surface of Livarot cheese using culture-dependent and independent approaches. Int. J. Food Microbiol. 133, 31–37. 10.1016/j.ijfoodmicro.2009.04.020, PMID: 19481828

[ref104] Mounier J. Monnet C. Vallaeys T. Arditi R. Sarthou A. S. Hélias A.. (2008). Microbial interactions within a cheese microbial community. Appl. Environ. Microbiol. 74:172. 10.1128/AEM.01338-07, PMID: 17981942 PMC2223212

[ref105] Mucchetti G. Bonvini B. Remagni M. C. Ghiglietti R. Locci F. Barzaghi S.. (2008). Influence of cheese-making technology on composition and microbiological characteristics of Vastedda cheese. Food Control 19, 119–125. 10.1016/j.foodcont.2007.02.011

[ref106] Neviani E. Bottari B. Lazzi C. Gatti M. (2013). New developments in the study of the microbiota of raw-milk, long-ripened cheeses by molecular methods: the case of grana Padano and Parmigiano Reggiano. Front. Microbiol. 4:36. 10.3389/fmicb.2013.00036, PMID: 23450500 PMC3584316

[ref107] Nicaud J. M. (2012). Yarrowia lipolytica. Yeast 29, 409–418. 10.1002/yea.2921, PMID: 23038056

[ref108] Nielsen K. F. Dalsgaard P. W. Smedsgaard J. Larsen T. O. (2005). Andrastins a–d, *Penicillium roqueforti* metabolites consistently produced in blue-mold-ripened cheese. J. Agric. Food Chem. 53, 2908–2913. 10.1021/jf047983u, PMID: 15826038

[ref109] Ozturkoglu-Budak S. Wiebenga A. Bron P. A. Vries R. P. D. (2016). Protease and lipase activities of fungal and bacterial strains derived from an artisanal raw ewe’s milk cheese. Int. J. Food Microbiol. 237, 17–27. 10.1016/j.ijfoodmicro.2016.08.007, PMID: 27541978

[ref110] Padilla B. Belloch C. López-Díez J. J. Flores M. Manzanares P. (2014). Potential impact of dairy yeasts on the typical flavour of traditional ewes’ and goats’ cheeses. Int. Dairy J. 35, 122–129. 10.1016/j.idairyj.2013.11.002

[ref111] Panikuttira B. O’Shea N. Tobin J. T. Tiwari B. K. O’Donnell C. P. (2018). Process analytical technology for cheese manufacture. Int. J. Food Sci. Technol. 53, 1803–1815. 10.1111/ijfs.13806

[ref112] Pino A. Liotta L. Randazzo C. L. Todaro A. Mazzaglia A. De Nardo F.. (2018). Polyphasic approach to study physico-chemical, microbiological and sensorial characteristics of artisanal Nicastrese goat’s cheese. Food Microbiol. 70, 143–154. 10.1016/j.fm.2017.09.005, PMID: 29173621

[ref113] Plutowska B. Wardencki W. (2007). Aromagrams – aromatic profiles in the appreciation of food quality. Food Chem. 101, 845–872. 10.1016/j.foodchem.2005.12.028

[ref114] Price E. J. Linforth R. S. T. Dodd C. E. R. Phillips C. A. Hewson L. Hort J.. (2014). Study of the influence of yeast inoculum concentration (*Yarrowia lipolytica* and *Kluyveromyces lactis*) on blue cheese aroma development using microbiological models. Food Chem. 145, 464–472. 10.1016/j.foodchem.2013.08.081, PMID: 24128502

[ref115] Rantsiou K. Urso R. Dolci P. Comi G. Cocolin L. (2008). Microflora of feta cheese from four Greek manufacturers. Int. J. Food Microbiol. 126, 36–42. 10.1016/j.ijfoodmicro.2008.04.031, PMID: 18555549

[ref116] Ray R. El Sheikha A. F. Kumar S. (2014). “Oriental fermented functional (probiotic) foods,” in Microorganisms and Fermentation of Traditional Foods. eds. Ray R. C. Montet D. (Boca Raton, Florida, USA: Science Publishers Inc. and CRC Press), 283–311.

[ref117] Rea M. C. Görges S. Gelsomino R. Brennan N. M. Mounier J. Vancanneyt M.. (2007). Stability of the biodiversity of the surface consortia of Gubbeen, a red-smear cheese. J. Dairy Sci. 90, 2200–2210. 10.3168/jds.2006-377, PMID: 17430918

[ref118] Reif G. D. Shahani K. M. Vakil J. R. Crowe L. K. (1976). Factors affecting b-complex vitamin content of cottage cheese1, 2. J. Dairy Sci. 59, 410–415. 10.3168/jds.S0022-0302(76)84221-X

[ref119] Rilla N. Martínez B. Delgado T. Rodríguez A. (2003). Inhibition of clostridium tyrobutyricum in Vidiago cheese by *Lactococcus lactis* ssp. lactis IPLA 729, a nisin Z producer. Int. J. Food Microbiol. 85, 23–33. 10.1016/S0168-1605(02)00478-6, PMID: 12810268

[ref120] Roostita R. Fleet G. H. (1996). The occurrence and growth of yeasts in camembert and blue-veined cheeses. Int. J. Food Microbiol. 28, 393–404. 10.1016/0168-1605(95)00018-6, PMID: 8652347

[ref121] Sandine W. E. Elliker P. R. (1970). Microbially induced flavors and fermented foods. Flavor in fermented dairy products. J. Agric. Food Chem. 18, 557–562. 10.1021/jf60170a023

[ref122] Santiago-López L. Aguilar-Toalá J. E. Hernández-Mendoza A. Vallejo-Cordoba B. Liceaga A. M. González-Córdova A. F. (2018). Invited review: bioactive compounds produced during cheese ripening and health effects associated with aged cheese consumption. J. Dairy Sci. 101, 3742–3757. 10.3168/jds.2017-13465, PMID: 29477517

[ref123] Schwenninger S. M. Meile L. Lacroix C. (2011). “Protective cultures, antimicrobial metabolites and bacteriophages for food and beverage biopreservation,” in 2—Antifungal Lactic Acid Bacteria and Propionibacteria for Food Biopreservation. ed. Lacroix C. (New York: Woodhead Publishing), 27–62.

[ref124] Seiler H. Busse M. (1990). The yeasts of cheese brines. Int. J. Food Microbiol. 11, 289–303. 10.1016/0168-1605(90)90022-W, PMID: 2282287

[ref125] Sert D. Akin N. Aktumsek A. (2014). Lipolysis in Tulum cheese produced from raw and pasteurized goats’ milk during ripening. Small Rumin. Res. 121, 351–360. 10.1016/j.smallrumres.2014.06.006

[ref126] Singh T. K. Drake M. A. Cadwallader K. R. (2003). Flavor of Cheddar cheese: a chemical and sensory perspective. Compr. Rev. Food Sci. Food Saf. 2, 166–189. 10.1111/j.1541-4337.2003.tb00021.x, PMID: 33451230

[ref127] Smit G. Smit B. A. Engels W. J. M. (2005). Flavour formation by lactic acid bacteria and biochemical flavour profiling of cheese products. FEMS Microbiol. Rev. 29, 591–610. 10.1016/j.fmrre.2005.04.002, PMID: 15935512

[ref128] Soggiu A. Piras C. Mortera S. L. Alloggio I. Urbani A. Bonizzi L.. (2016). Unravelling the effect of clostridia spores and lysozyme on microbiota dynamics in grana Padano cheese: a metaproteomics approach. J. Proteome 147, 21–27. 10.1016/j.jprot.2016.03.035, PMID: 27045942

[ref129] Sørensen L. M. Gori K. Petersen M. A. Jespersen L. Arneborg N. (2011). Flavour compound production by *Yarrowia lipolytica*, *Saccharomyces cerevisiae* and *Debaryomyces hansenii* in a cheese-surface model. Int. Dairy J. 21, 970–978. 10.1016/j.idairyj.2011.06.005

[ref130] Spano G. Russo P. Lonvaud-Funel A. Lucas P. Alexandre H. Grandvalet C.. (2010). Biogenic amines in fermented foods. Eur. J. Clin. Nutr. 64, S95–S100. 10.1038/ejcn.2010.218, PMID: 21045859

[ref131] Steensels J. Verstrepen K. J. (2014). Taming wild yeast: potential of conventional and nonconventional yeasts in industrial fermentations. Annu. Rev. Microbiol. 68, 61–80. 10.1146/annurev-micro-091213-113025, PMID: 24773331

[ref132] Suzuki-Iwashima A. Matsuura H. Iwasawa A. Shiota M. (2020). Metabolomics analyses of the combined effects of lactic acid bacteria and *Penicillium camemberti* on the generation of volatile compounds in model mold-surface-ripened cheeses. J. Biosci. Bioeng. 129, 333–347. 10.1016/j.jbiosc.2019.09.005, PMID: 31611057

[ref133] Suzzi G. Lombardi A. Lanorte M. T. Caruso M. Andrighetto C. Gardini F. (2000). Phenotypic and genotypic diversity of yeasts isolated from water-buffalo mozzarella cheese. J. Appl. Microbiol. 88, 117–123. 10.1046/j.1365-2672.2000.00926.x, PMID: 10735250

[ref134] Tajammal Munir M. Yu W. Young B. R. Wilson D. I. (2015). The current status of process analytical technologies in the dairy industry. Trends Food Sci. Technol. 43, 205–218. 10.1016/j.tifs.2015.02.010

[ref135] Talbot-Walsh G. Kannar D. Selomulya C. (2018). A review on technological parameters and recent advances in the fortification of processed cheese. Trends Food Sci. Technol. 81, 193–202. 10.1016/j.tifs.2018.09.023

[ref136] Tedeschi T. Galaverna G. Dossena A. Sforza S. (2013). “Chapter 19: Comprehensive analytical chemistry,” in Cheeses. eds. de la Guardia M. Gonzálvez A. (Italy: Elsevier), 479–509.

[ref137] Temizkan R. Yasar K. Hayaloglu A. A. (2015). Changes during ripening in chemical composition, proteolysis, volatile composition and texture in Kashar cheese made using raw bovine, ovine or caprine milk. Int. J. Food Sci. Technol. 49, 2643–2649. 10.1111/ijfs.12597

[ref138] Thierry A. Collins Y. F. Abeijón Mukdsi M. C. McSweeney P. L. H. Wilkinson M. G. Spinnler H. E. (2017). “Chapter 17: Lipolysis and metabolism of fatty acids in cheese,” in Cheese. 4th *Edn*. eds. McSweeney P. L. H. Fox P. F. Cotter P. D. Everett D. W. (San Diego: Academic Press), 423–444.

[ref139] Tomita S. Nakamura T. Okada S. (2018). NMR- and GC/MS-based metabolomic characterization of sunki, an unsalted fermented pickle of turnip leaves. Food Chem. 258, 25–34. 10.1016/j.foodchem.2018.03.038, PMID: 29655730

[ref140] Upreti P. Metzger L. E. (2006). Influence of calcium and phosphorus, lactose, and salt-to-moisture ratio on Cheddar cheese quality: manufacture and composition. J. Dairy Sci. 89, 420–428. 10.3168/jds.S0022-0302(06)72106-3, PMID: 16428612

[ref141] Urbach G. (1993). Relations between cheese flavour and chemical composition. Int. Dairy J. 3, 389–422. 10.1016/0958-6946(93)90025-U

[ref142] Vedamuthu E. R. (1994). The dairy *Leuconostoc*: use in dairy products. J. Dairy Sci. 77, 2725–2737.

[ref143] Viljoen B. C. Khoury A. R. Hattingh A. (2003). Seasonal diversity of yeasts associated with white-surface mould-ripened cheeses. Food Res. Int. 36, 275–283. 10.1016/S0963-9969(02)00169-2

[ref144] Vlieg J. V. H. Hugenholtz J. (2007). Mining natural diversity of lactic acid bacteria for flavour and health benefits. Int. Dairy J. 17, 1290–1297. 10.1016/j.idairyj.2007.02.010

[ref145] Voigt D. D. Chevalier F. Donaghy J. A. Patterson M. F. Qian M. C. Kelly A. L. (2012). Effect of high-pressure treatment of milk for cheese manufacture on proteolysis, lipolysis, texture and functionality of Cheddar cheese during ripening. Innovative Food Sci. Emerg. Technol. 13, 23–30. 10.1016/j.ifset.2011.10.004

[ref146] Wang C. L. Shi D. J. Gong G. L. (2008). Microorganisms in Daqu: a starter culture of Chinese Maotai-flavor liquor. World J. Microbiol. Biotechnol. 24, 2183–2190. 10.1007/s11274-008-9728-0

[ref147] Wolfe B. E. Button J. E. Santarelli M. Dutton R. J. (2014). Cheese rind communities provide tractable systems for in situ and in vitro studies of microbial diversity. Cell 158, 422–433. 10.1016/j.cell.2014.05.041, PMID: 25036636 PMC4222527

[ref148] Woo A. H. Kollodge S. Lindsay R. C. (1984). Quantification of major free fatty acids in several cheese varieties1. J. Dairy Sci. 67, 874–878. 10.3168/jds.S0022-0302(84)81380-6

[ref149] Woo A. H. Lindsay R. C. (1984). Concentrations of major free fatty acids and flavor development in italian cheese varieties1. J. Dairy Sci. 67, 960–968. 10.3168/jds.S0022-0302(84)81394-6

[ref150] Wu Q. Xu Y. Chen L. (2012). Diversity of yeast species during fermentative process contributing to Chinese Maotai-flavour liquor making. Lett. Appl. Microbiol. 55, 301–307. 10.1111/j.1472-765X.2012.03294.x, PMID: 22862564

[ref151] Xiao J. Chen Y. Li J. Shi X. Deng L. Wang B. (2020). Evaluation of the effect of auxiliary starter yeasts with enzyme activities on Kazak cheese quality and flavor. Front. Microbiol. 11:614208. 10.3389/fmicb.2020.614208, PMID: 33391244 PMC7772356

[ref152] Zerfiridis G. K. Vafopoulou-Mastrogiannaki A. Litopoulou-Tzanetaki E. (1984). Changes during ripening of commercial Gruyère cheese. J. Dairy Sci. 67, 1397–1405. 10.3168/jds.S0022-0302(84)81454-X

[ref153] Zheng X. C. Ge Z. Lin K. Zhang D. Chen Y. Xiao J.. (2021). Dynamic changes in bacterial microbiota succession and flavour development during milk fermentation of Kazak artisanal cheese. Int. Dairy J. 113:104878. 10.1016/j.idairyj.2020.104878

[ref154] Zheng X. J. Li K. Shi X. Ni Y. Li B. Zhuge B. (2018a). Potential characterization of yeasts isolated from Kazak artisanal cheese to produce flavoring compounds. MicrobiologyOpen 7:e00533. 10.1002/mbo3.533 PMC5822340 29277964

[ref155] Zheng X. J. Liu F. Shi X. Wang B. Li K. Li B.. (2018b). Dynamic correlations between microbiota succession and flavor development involved in the ripening of Kazak artisanal cheese. Food Res. Int. 105, 733–742. 10.1016/j.foodres.2017.12.007 29433268

[ref156] Zhu J. Chen F. Wang L. Niu Y. Yu D. Shu C.. (2015). Comparison of aroma-active volatiles in oolong tea infusions using GC–olfactometry, GC–FPD, and GC–MS. J. Agric. Food Chem. 63, 7499–7510. 10.1021/acs.jafc.5b02358, PMID: 26257073

